# The emergence of eukaryotes as an evolutionary algorithmic phase transition

**DOI:** 10.1073/pnas.2422968122

**Published:** 2025-03-27

**Authors:** Enrique M. Muro, Fernando J. Ballesteros, Bartolo Luque, Jordi Bascompte

**Affiliations:** ^a^Institute of Organismic and Molecular Evolution, Johannes Gutenberg University of Mainz, Mainz DE-55128, Germany; ^b^Astronomical Observatory, University of Valencia, Paterna, Valencia E-46980, Spain; ^c^Department of Applied Mathematics and Statistics, Escuela Técnica Superior de Ingeniería Aeronáutica y del Espacio, Universidad Politécnica de Madrid, Madrid E-28040, Spain; ^d^Department of Evolutionary Biology and Environmental Studies, University of Zurich, Zurich CH-8057, Switzerland

**Keywords:** eukaryotic cell, scaling law, gene length, protein length

## Abstract

The origin of eukaryotes represents one of the most significant events in evolution since it allowed the posterior emergence of multicellular organisms. Yet, it remains unclear how existing regulatory mechanisms of gene activity were transformed to allow this increase in complexity. Here, we address this question by analyzing the length distribution of proteins and their corresponding genes for 6,519 species across the tree of life. We find a scale-invariant relationship between gene mean length and variance maintained across the entire evolutionary history. Using a simple model, we show that this scale-invariant relationship naturally originates through a simple multiplicative process of gene growth. During the first phase of this process, corresponding to prokaryotes, protein length follows gene growth. At the onset of the eukaryotic cell, however, mean protein length stabilizes around 500 amino acids. While genes continued growing at the same rate as before, this growth primarily involved noncoding sequences that complemented proteins in regulating gene activity. Our analysis indicates that this shift at the origin of the eukaryotic cell was due to an algorithmic phase transition equivalent to that of certain search algorithms triggered by the constraints in finding increasingly larger proteins.

## Significance

For almost half the history of life on Earth, the complexity of all organisms was limited to that of simple prokaryotic cells such as contemporary bacteria. The process by which genes are activated, which is at the root of the functioning of all living beings, was entirely regulated by proteins. This set up a limit on cellular complexity, as finding even larger proteins became computationally unfeasible. The eukaryotic cell—characterized by membrane-bound nucleus and organelles—emerged as a compromise between a conserved process of gene growth and a change in genetic regulation, which incorporated noncoding sequences. This increase in cellular complexity, which occurred continuously but in an abrupt manner at a critical point, unlocked the path toward multicellular organisms.

The history of life on Earth has been punctuated by several major transitions, among which the origin of the eukaryotic cell is particularly relevant (1, 2). For much of the history of life, organisms were confined to the simple, undifferentiated prokaryotic cell represented by Bacteria and Archaea (3–5). The emergence of the eukaryotic cell, most likely arising from the symbiosis between two previously unrelated organisms—an archaeon host cell and a bacterium that would become mitochondria (6–8)—brought a new cellular structure with membrane-bound nucleus and organelles. Without this evolutionary event, the posterior evolution of multicellular organisms represented by animals, land plants, and the majority of fungi would not have been possible.

This increase in cellular complexity required new and more sophisticated mechanisms of gene regulation provided by noncoding regions such as introns (9, 10). It is not clear, however, which balance of novelty and continuity in the underlying mechanisms of gene and protein evolution allowed this major reorganization of life. For example, had a protein-based genetic regulation reached a limit? And if so, how did the relationship between gene and protein length change beyond that? We address these questions by studying some aggregate properties of protein-coding genes (genes hereafter) and their corresponding proteins from a critical phenomena approach (11).

We start by analyzing the length distribution of genes and proteins, and by modeling the basic mechanism of gene growth pointed out by these patterns. This will lead us to a series of predictions on the process of gene growth through time, the relationship between mean gene length and variance, and that between mean gene and protein lengths, which we will contrast with empirical data. Our blend of theoretical and empirical approaches will ultimately uncover how the tension between a conserved process of gene growth and the constraints on the increasingly longer proteins resolved in a phase transition signaling the emergence of the eukaryotic cell.

## Results

### Gene Length Distributions.

**Fig. 1.**
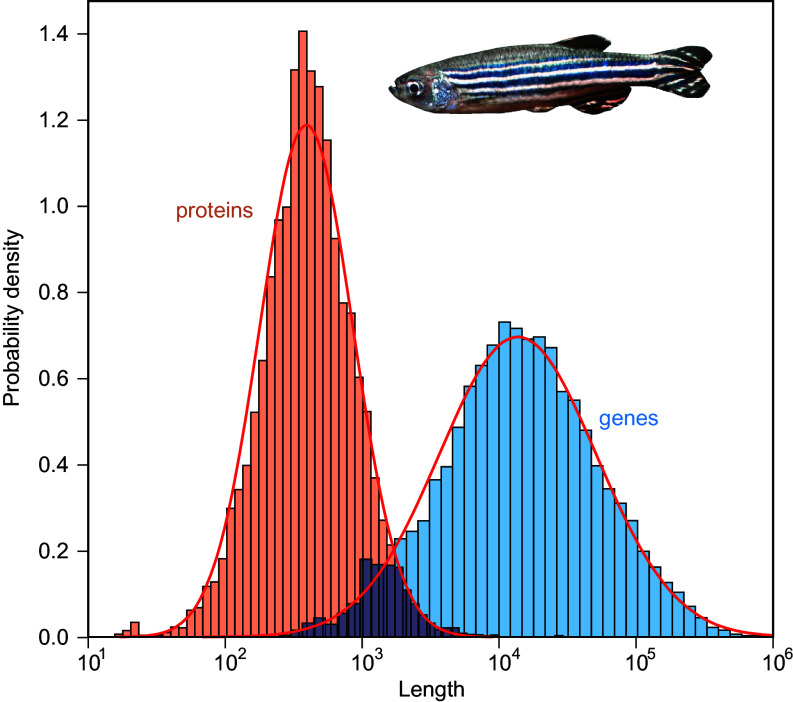
Gene and protein length distributions are lognormal. Length distributions for genes (blue, measured in number of base pairs) and their corresponding proteins (orange, measured in number of amino acids) for *Danio rerio* (zebrafish). Note that lengths are represented on a logarithmic scale. The red curves are fits to lognormal distributions. Mean lengths (and SDs) of genes and proteins are 31,084 (51,637) base pairs and 538 (562) amino acids, respectively. Total number of genes and proteins plotted is 25,432 and 25,706, respectively. These lognormal distributions are typically found in almost all species across the tree of life (*Materials and Methods*).

We begin by characterizing the length distribution of genes (measured as number of nucleotides) for 33,627 annotated genomes extracted from the Ensembl database (12). This includes almost 150 million genes from a broad range of organisms including Archaea, Bacteria, protists, Fungi, plants, invertebrates, and vertebrates. Fig. 1 (in blue) illustrates this distribution for the example of the zebrafish (*Danio rerio*). The lognormal distribution provides the best fit to the gene length distributions in the large majority of the species analyzed (*Materials and Methods* and *SI Appendix*, Figs. S1 and S2, *Left*). Although previous work analyzing protein length distributions emphasized their universality (13) or found evidence for both gamma and lognormal distributions in small datasets (14–16), there was no previous information regarding gene distributions at such a large scale.

What insights can be gleaned about the evolution of genes from this observation? It is well known that lognormal distributions, as a consequence of the Central Limit Theorem (15, 17), are a typical outcome of multiplicative processes. Also, several mechanisms of genomic growth are multiplicative processes that modify the length of a gene by a stochastic factor. Examples include gene elongation (18), tandem duplication (19, 20), or total/partial genomic duplication (21, 22). Consequently, it is reasonable to model gene growth as a multiplicative stochastic process.

### A Model of Gene Growth.

We have developed a simple multiplicative model of gene growth. Essentially, we start with a series of genes with arbitrary initial lengths. We then iterate a process by which a gene is randomly chosen and its length is multiplied by a random number *ζ* generated from a given probability density function (*SI Appendix*). As expected, this model leads to lognormal gene length distributions. This result is robust even when additional additive processes of genome growth are also considered, as the geometric growth dominates over the arithmetic one (*SI Appendix*).

More importantly, our model predicts two laws describing the growth of genes through time, validated by simulations (on average; individual realizations depend on their history). First, mean gene length ⟨L⟩ grows exponentially through time according to:

[1] $$\begin{array}{c} \langle L(t)\rangle =L_{0}\cdot \exp((\left\langle \zeta \right\rangle -1)\cdot t), \end{array}$$

where *L*_0_ stands for the mean gene length of the Last Universal Common Ancestor (LUCA) and ⟨ζ⟩ is the mean of the stochastic multiplicative factor. This predicted exponential growth is compatible with previous empirical findings reporting that genes increase in length over evolutionary time, with a potential acceleration in the most recent ones (23).

Second, the mean of the logarithm of gene lengths ⟨logL⟩ (mean gene log length hereafter) grows linearly through time according to:

[2] $$\begin{array}{c} \left\langle \log L\left(t\right)\right\rangle =G_{0}+\left\langle \log\zeta \right\rangle \cdot t, \end{array}$$

where *G*_0_ stands for the mean gene log length of LUCA’s genome, and ⟨logζ⟩ is the mean of the logarithm of the stochastic multiplicative factor.

In order to corroborate our two laws of gene growth (Eqs. **1** and **2**), we have calculated the average mean gene lengths and mean gene log lengths for different phyletic groups. We have then plotted these values against their divergence time with *Homo sapiens* measured from LUCA (*Materials and Methods*). The obtained results are in good agreement with our model’s predictions ($R^{2}=0.79$, *SI Appendix*, Fig. S3). Additionally, we also find good empirical support for the linear relationship between log⟨L⟩ and ⟨logL⟩ that naturally emerges from our two laws of gene growth ($R^{2}=0.97$; *SI Appendix*, Eq. **3** and Fig. S4).

By combining our predicted laws of gene growth and the observed lognormal gene distributions described in the previous section, one can derive the following scaling law linking mean gene length and variance ($\sigma^{2}$) (*SI Appendix*):

[3] $$\begin{array}{c} \left\langle L^{2}\right\rangle =\sigma^{2}+{\left\langle L\right\rangle }^{2}=a\cdot {\left\langle L\right\rangle }^{\beta }, \end{array}$$

where $\left\langle L^{2}\right\rangle$ stands for the second raw moment of gene lengths, its asymptotic limit for ⟨L⟩≫1 being:

[4] $$\begin{array}{c} \sigma^{2}≃a\cdot {\left\langle L\right\rangle }^{\beta }, \end{array}$$

and where parameters *a* and *β* are completely determined by those of the two gene growth laws according to:

[5] $$\begin{array}{c} \beta =4-\frac{2\langle \log\zeta \rangle }{\langle \zeta \rangle -1},\;a=\frac{{L_{0}}^{4-\beta }}{\exp\left({2G}_{0}\right)}. \end{array}$$

### A Scaling Law for Gene Growth.

**Fig. 2.**
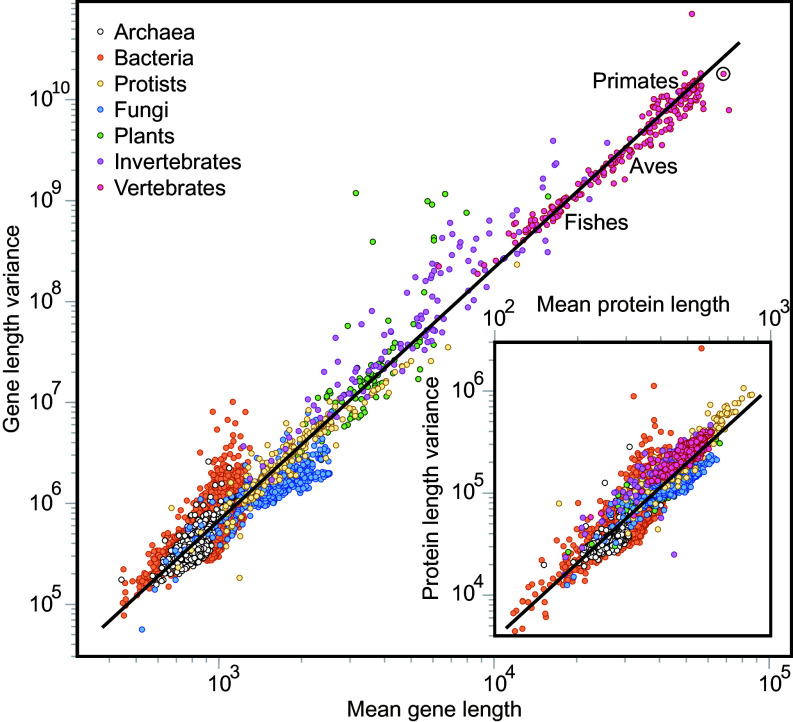
Scale-invariant relationship between mean gene length and variance. Each dot in this log–log plot represents the genome of a single species (n= 33,627), with *H. sapiens* highlighted by the circle. Gene length is measured in number of base pairs. Different colors identify major phyletic groups. As a guide to the eye, we have added the labels fishes, Aves, and Primates, marking the region where these groups cluster. The line represents the best fit to a power law (Eq. **4**; $\sigma^{2}=0.0159\left(\pm 0.0004\right){\left\langle L\right\rangle }^{2.511(\pm 0.004)}$, $R^{2}=0.92$). *Inset*: equivalent representation for protein length (measured in number of amino acids) for 9,913 species. The line represents the best fit to an equivalent power law (*SI Appendix*; $\sigma_{p}^{2}=0.0168\left(\pm 0.0015\right){\left\langle L_{p}\right\rangle }^{2.605(\pm 0.016)}$, $R^{2}=0.73$). Major phyletic groups appear sequentially across the scaling law for genes, but not so clearly in the equivalent for proteins.

In order to contrast our predicted scaling law with empirical evidence, we plot the mean and variance of the 33,627 gene length distributions. We recover a scaling law covering more than two orders of magnitude (Fig. 2 and *SI Appendix*, Fig. S5 for the second raw moment). Fitting these data to Eq. **3** yields *β* = 2.29 and *a* = 0.21 with $R^{2}=0.98$. These two values determine the relationship between ⟨logζ⟩ and ⟨ζ⟩, and between *L*_0_ and *G*_0_, through Eq. **5**. Thus, by introducing one value of each pair we can estimate the other one, a procedure that gives numbers consistent with those coming from the fits of the two growth laws (*SI Appendix*, Fig. S3). This procedure leads to $L_{0}=554$, $G_{0}=6.18$, ⟨ζ⟩=1.00101, and ⟨logζ⟩=0.00087 as final estimations.

Overall, these results show how the observed relationship between mean gene length and variance emerges from basic multiplicative processes of gene growth. In ecology, an equivalent scaling law relating mean animal population density and its associated variance is referred to as Taylor’s law (24). Similar relationships have been described in biology, although in different contexts than the one here described (25–27).

The above relationship between mean and variance is scale-invariant. Its slope is constant across a wide range of gene sizes. This indicates that the multiplicative process of gene growth has been maintained throughout the entire evolutionary history, with at least the same mean and mean log of the stochastic multiplicative factor, ⟨ζ⟩ and ⟨logζ⟩, respectively. Importantly, we can see the trace of evolution on Taylor’s law. Different phyletic groups are indeed clustered following the order of evolutionary origin, from Archaea and Bacteria at the *Bottom Left* corner, to vertebrates at the *Top Right* corner (Fig. 2). This evolutionary unfolding of the different phyletic groups across Taylor’s law is a direct consequence of the laws of gene growth through time plotted in *SI Appendix*, Fig. S3, as higher ranges of gene length were reached more recently. This depicts an increase of organismal complexity across the scale-invariant mechanism of gene growth through evolution (10, 28). We find that mean gene length, therefore, is a good surrogate of organismal complexity (*SI Appendix*, Fig. S6, *Left*). It is also a robust metric, as it is rather insensitive to incomplete data. On one hand, our finding is in agreement with previous work showing that mean gene length is shorter for prokaryotes than eukaryotes (29). On the other hand, our result expands previous work exploring alternative metrics such as number of genes or DNA content, which have been proven to show poorer correlations with organismal complexity or are more sensitive to sample size (30).

### Patterns in Protein Length Distributions.

So far, we have analyzed genes. Regarding proteins, we have studied the distributions of 9,913 reference proteomes from Uniprot (31), including up to a total of 55 million proteins. From this analysis, we recover our two major findings for genes. First, protein length distributions are also lognormal across the whole tree of life, as Fig. 1 (orange) illustrates for the example of *D. rerio* (*Materials and Methods* and *SI Appendix*, Fig. S2, *Right*).

Second, mean protein length $\left\langle L_{p}\right\rangle$ and its associated variance $\sigma_{p}^{2}$ also conform to a Taylor’s law, but with a narrower range in mean protein length (Fig. 2, *Inset* and *SI Appendix*, Fig. S5, *Insets*). This may not be surprising for species such as Archaea or Bacteria (32), given that their genes are translated into proteins in proportion 3:1 (*SI Appendix*, Fig. S7, *Left*) and, therefore, the multiplicative process of genes tows the statistics of proteins. But this is not the case for species with gene noncoding sequences (*SI Appendix*, Fig. S7, *Right*), for which the 3:1 relationship breaks down. Also, the evolutionary order of the different phyletic groups across Taylor’s law for genes is blurred for proteins, as particularly appreciated by the overlap of recent groups at the higher end of the distribution (Fig. 2, *Inset*). Indeed, proteins do not follow equivalent growth laws through much of the evolutionary process as at a certain point, protein distributions were decoupled from the multiplicative process of genes. Thus, contrary to genes, mean protein length does not seem to be as good as a proxy for organismal complexity (*SI Appendix*, Fig. S6, *Right*).

It has been recently discussed that differences in protein length could be explained by annotation artifacts. This is most likely the case for some genomes corresponding to atypical protein length distributions characterized by a dominance of small proteins (13). However, this seems the exception to a general pattern by which protein length distribution is remarkably similar across the tree of life, with functional constraints such as cost of protein synthesis and speed of protein folding limiting the upper end of their distribution (13). One strength of our approach is focusing on the mean protein length within an organism, which as noted above reduces the amount of noise introduced by potential annotation artifacts in a given protein.

### Relation Between Gene and Protein Lengths.

Having considered genes and proteins independently, we now proceed to compare their mean lengths. For this, we have cross-correlated the lists of genomes and proteomes (*Materials and Methods*), obtaining 6,519 organisms for which we have records in both datasets.

Genes are formed by coding (CDS) and noncoding (nCDS) sequences— the latter constituted by untranslated regions and introns (9)—(*SI Appendix*, Fig. S8, *Bottom*). There are other types of noncoding regions outside genes involved in gene regulation, and yet here we focus on those located within genes for two reasons. First, introns play a more significant role in gene regulation, for example through alternative splicing (9, 10). Second, DNA regions that encode proteins are the best-annotated elements due to their high conservation across species. Similarly, introns, as complementary regions, are also well annotated.

**Fig. 3.**
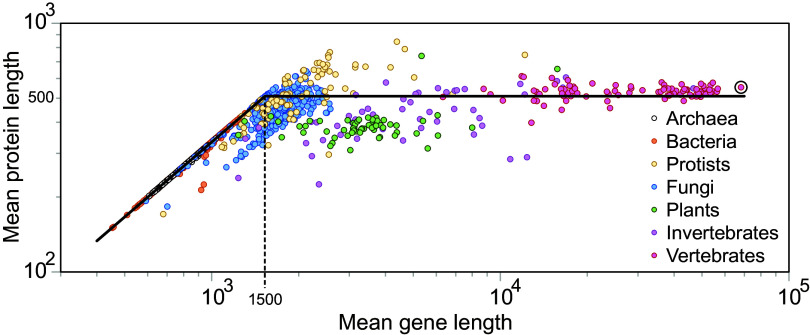
Threshold in the relationship between mean protein and gene lengths. Each dot represents a single species (n= 6,519) (see *Merging Ensembl and Uniprot Annotations* for details on data selection), with *H. sapiens* highlighted by the circle. Gene and protein lengths are measured in number of base pairs and amino acids, respectively. Different colors identify major phyletic groups. The black line (Eq. **6**) describes the trend observed in the data separating two distinct phases. In the first phase, mean protein length grows proportionally to mean gene length. Beyond a critical mean gene length around 1,500 base pairs, mean protein size stabilizes and is not any more a function of mean gene length (*SI Appendix*, Fig. S9, *Bottom*).

Given the aforementioned two components of a given gene *i*, we can write its length as $L_{i}=l_{i}+{\overset{¯}{l}}_{i}$, where *l* stands for CDS and $\overset{¯}{l}$ for nCDS. When plotting mean gene length ⟨L⟩ versus mean protein length $\left\langle L_{p}\right\rangle$ (Fig. 3), the expected linear relationship $\langle L\rangle =\langle l\rangle =3\cdot \left\langle L_{p}\right\rangle$ corresponding to the case when $\langle \overset{¯}{l}\rangle =0$ can be observed clearly for Archaea and Bacteria (and also for some protists and many unicellular fungi). But beyond a critical mean gene length *L*_*c*_ around 1,500 base pairs (*Materials and Methods* and *SI Appendix*, Fig. S9), mean protein length remains constant around $L_{c}/3$. This is in agreement with a recent finding showing that protein distributions are very similar across species (13). Beyond this point, genes grow by including mainly only nCDS. So, if we assume as a first approximation that $\left\langle L_{p}\right\rangle \approx L_{c}/3$ for mean gene lengths larger than *L*_*c*_, we can describe this behavior as a threshold phenomenon:

[6] $$\begin{array}{c} \left\langle L_{p}\right\rangle \approx \left\{\begin{array}{ccc} \frac{1}{3}\left\langle L\right\rangle & \text{if} & \left\langle L\right\rangle \leq L_{c}\\ \frac{1}{3}L_{c} & \text{if} & \left\langle L\right\rangle >L_{c} \end{array}\right. \end{array}$$

The ratio $\left\langle l\right\rangle /\left\langle L\right\rangle =3\cdot \left\langle L_{p}\right\rangle /\left\langle L\right\rangle$ represents, on average, the mean fraction of coding sequences within a gene. Then $\rho \equiv 1-3\cdot \left\langle L_{p}\right\rangle /\langle L\rangle$ represents the fraction of gene noncoding sequences. In Bacteria and Archaea, $\langle L\rangle =3\cdot \left\langle L_{p}\right\rangle$ and therefore *ρ* = 0, while in eukaryotes ρ∈(0,1). We can obtain the fraction of gene noncoding sequences as a function of mean gene length from Eq. **6**:

[7] $$\begin{array}{c} \rho \left(\langle L\rangle \right)\approx \left\{\begin{array}{ccc} 0 & \text{if} & \langle L\rangle \leq L_{c}\\ 1-L_{c}/\langle L\rangle & \text{if} & \langle L\rangle >L_{c} \end{array}\right. \end{array}$$

**Fig. 4.**
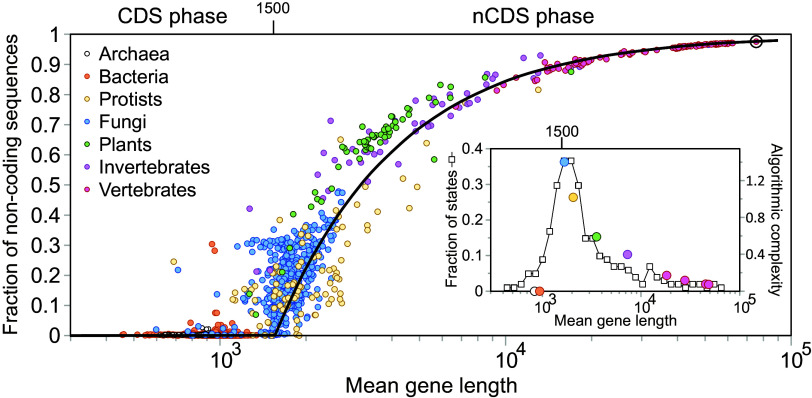
Second-order phase transition in the fraction of gene noncoding sequences. Each dot represents a single species (n= 6,519), with *H. sapiens* highlighted by the circle. Gene length is measured in number of base pairs. Different colors identify major phyletic groups. The black line represents Eq. **7** assuming a critical mean gene length of 1,500 base pairs separating the coding sequence (CDS) and noncoding sequence (nCDS) phases. *Inset*: White squares describe the scatter of values for the fraction of gene noncoding sequences (i.e., fraction of states) at a given mean gene length, as described in *Materials and Methods*; colored points represent the experimental algorithmic complexity $\tau_{\text{exp}}$ for the groups of organisms shown in *SI Appendix*, Fig. S3. This is estimated as the quotient of their divergence time with *H. sapiens*, $t_{\text{div}}$ (in million years, obtained from TimeTree.org), measured from LUCA (3,600 My ago) (5), divided by the average mean gene length of the group, *L*_*gr*_, in base pairs: $\tau_{\text{exp}}=\left(3,600-t_{\text{div}}\right)/L_{\mathrm{gr}}$. Both measures become maximal around the threshold 1,500 base pairs.

Eq. **7** fits quite well our data, separating species in two phases (Fig. 4, black line). For mean gene lengths below $L_{c}=1,500$, the fraction of gene noncoding sequences is practically zero (*ρ* < 0.1 for 98.9% of the species). Genes in this region are dominated by coding sequences. Prokaryotes (Bacteria and Archaea) can be found exclusively here. Beyond *L*_*c*_, we are in the noncoding-sequence phase, where *ρ* > 0 (only 1.1% of the organisms have *ρ* < 0.1). Now the fraction of gene noncoding sequences grows with species complexity roughly following the second part of Eq. **7**. This is the phase where we encounter all animals and most plants and fungi.

### A Phase Transition at a Critical Gene Length.

Our reported threshold in the fraction of gene noncoding sequences for a critical mean gene length has the typical appearance of a second-order phase transition (11). In the context of our results, this means that the transition from genes containing exclusively coding sequences to genes containing a fraction of noncoding sequences is abrupt and yet continuous.

A general phenomenon associated to second-order phase transitions is critical slowing down: The system becomes increasingly slow in recovering from small perturbations as it approaches the critical point. As a consequence, the system tends to get trapped in metastable states, which leads to a higher scatter of states around the critical point (11, 33). This scatter can be measured by means of the fraction of states (*Materials and Methods*), which gives an estimation of how many contrasting *ρ*-values exist for a given mean gene length. As noted in the *Inset* of Fig. 4 (white squares), this fraction peaks around *L*_*c*_. This is also the case for the entropy of *ρ*, which although noisier, also shows a maximum around the same value of mean gene length (*SI Appendix*, Fig. S10). Another signal of critical slowing down can be found in the histogram of mean gene length for fungi (*SI Appendix*, Fig. S9, *Top*). These are indicators that we are facing a second-order phase transition, but what kind of phase transition?

### Algorithmic Easy–Hard–Easy Pattern.

A particular type of second-order phase transition is found in the performance of search algorithms used in NP-complete problems such as the traveling salesman (34, 35) or graph coloring (36). These algorithmic phase transitions show the so-called easy–hard–easy pattern (37). As the size of the problem—and therefore that of the search space—first increases, the time that the algorithm requires to find a solution starts growing, up to a point where it reaches a maximum. In the transition, the system shifts to another type of solution so that computational time now decreases as the size of the problem keeps increasing.

Although evolution does not strictly provide solutions, we can use the term in the previous algorithmic context of optimizing functions under certain constraints. Thus, we can think of evolution as a gigantic optimization search algorithm where each species provides its own approximation to the solution. This analogy is not new (38). Indeed, the process of natural selection has inspired one type of optimization evolutionary algorithms named genetic algorithms. Such evolutionary algorithms perform well in solving the types of problems mentioned above (39). Therefore, we ask ourselves whether we are facing an algorithmic phase transition.

In the context of such transitions, the algorithmic complexity (40) can be defined as the average time spent per size unit to reach a solution. This can be rephrased in our case as the time spent by evolution per nucleotide to reach a species characterized by a given mean gene length, $\tau =t/\langle L\rangle$ (*SI Appendix*). We can estimate this by dividing divergence times for different phyletic groups—taking *H. sapiens* as a reference—by their corresponding average mean gene lengths.

As shown in Fig. 4, *Inset* (round colored dots), the computational cost reaches a maximum of hardness around the critical point *L*_*c*_ and decreases as we move away from it. As expected in this kind of phase transitions, this peak corresponds to the point when evolution added a novel mechanism of gene regulation based on noncoding sequences. Note that this computational cost correlates very well with the fraction of states, despite both measures are obtained from different datasets.

By assuming we are facing an algorithmic phase transition we can furthermore deduce theoretically *L*_*c*_ as the point where *τ* becomes maximal, $d\tau /d\langle L\rangle =0$, which leads to $L_{c}=e\cdot L_{0}$ (*SI Appendix*). Solving for *L*_0_ from Eq. **5** right determines *L*_*c*_ as a function of parameters *a* and *β* of Taylor’s law and *G*_0_ of the mean gene log length growth law (Eq. **2**), resulting in $L_{c}≃1,500$ base pairs, which matches our previous results. Similarly solving for *t* in Eq. **1** and using $\left\langle L\right\rangle =L_{c}$ gives *t*_*c*_, the moment when the critical gene length was reached for the first time, as a function of parameter ⟨ζ⟩ of the mean gene length growth law (Eq. **1**) resulting in $t_{c}≃1,000$ My after LUCA. Assuming that ∼3,600 My have gone by since the emergence of LUCA (5), we can estimate that the critical point *L*_*c*_ was first reached approximately 2,600 My ago. Remarkably, this estimation is a figure close to that of the emergence of the eukaryotic cell, clearly within the chronology from the first eukaryotic common ancestor to the last eukaryotic common ancestor, around 2,900–2,300 My ago (41–46).

Overall, the assumption of an algorithmic phase transition naturally relates our three main results, namely, the laws of gene growth, the scale-invariant relationship between mean gene length and variance, and the phase transition in the relationship between mean gene and protein lengths. This signals how evolution has managed to combine a conserved mechanism of gene growth with the novelty that allowed escaping the computational limit.

## Discussion

The origin of the eukaryotic cell left a signal in terms of a second-order algorithmic phase transition, following a crisis of computational hardness. In the first phase, characterized by prokaryotes, all genes were translated into proteins through a conserved mechanism of protein-based genetic regulation: Here genes have a linear response as a gene with length *l* expresses a protein with length l/3. In this phase, genes are initially small. The search space is also small, thus making it easy to find a solution based on proteins. The sophistication of this regulatory mechanism could be enhanced by increasing the size of proteins, but only up to a point beyond which the search for new proteins became computationally unfeasible (47). Once this point was reached, mean eukaryotic gene length kept growing exponentially—as indicated by the scale-invariance of our reported Taylor’s law—but now incorporating almost exclusively noncoding sequences, in agreement with the intron-late hypothesis (32, 48). Now, a single gene can generate several proteins by means of alternative splicing and free RNAs with regulatory functions. As genes grew beyond this point, the system’s available solutions grew enormously due to the explosion of possible combinations. Finding one given solution, therefore, becomes gradually easier.

Other traditional approaches, such as theoretical population genetics, have attempted to explain the emergence of complexity. They emphasize the role of nonadaptive mechanisms such as genetic drift in leading to modifications that may or may not be fixed depending on population size. Unicellular species have such large population sizes that even advantageous modifications tend to be diluted. In multicellular species having much smaller population sizes, on the contrary, such modifications are easily fixed even if they are unfavorable (49). Our approach shares the emphasis on neutral, nonadaptive mechanisms as hypothesis for the origins of organismal complexity with population genetics. At the same time, however, it complements theory on population genetics in its prediction of a phase transition and its specific dependence on a critical mean gene length. Our approach, therefore, aligns with recent calls to complement population genetics given its assumption of a single relevant scale and its inability to make predictions on the phenotypic evolution of form (50).

Our portrait of a phase transition is in agreement with the lack of intermediate forms behind the emergence of eukaryotes—what has been termed a black hole at the heart of biology (51). Previous work has also highlighted the shift between prokaryotes and eukaryotes on the basis of energetic constraints (7, 8), or metabolic allocation (27). Our results add an algorithmic dimension to this view emphasizing the role of constraints. It reconciles the contingency of evolution—exemplified by the random exploration of the search space—and the universality of physics (52).

Importantly, our framework has an unparalleled predictive power, as shown by its ability to predict not only the specific laws governing the growth of genes and proteins across the entire evolutionary history, but also the precise moment in time at which eukaryotes emerged and the critical mean gene length at which this occurred. As with any approach, ours also suffers from some limitations. Chiefly among those, our analysis of average gene and protein length is based on samples of contemporary organism, a sort of “photo finish” from which we reconstruct the evolutionary history. Also, our model simplifies the evolutionary trajectory of a species to that of a random walk, thus disregarding important evolutionary mechanisms such as speciation.

Future work should explore the feedback between energy and information in explaining major transitions in evolution. For example, the noncoding phase here reported was only possible when a large fraction of mitochondrial genes were incorporated into the nuclear genome, which has been explained on the basis of energetic considerations (8). Similarly, it has been adduced that the differences in metabolic partitioning between prokaryotes and eukaryotes could be related to the higher variability in proteins for the same amount of transcriptional resources (27). The algorithmic phase transition here reported allows us to predict the mechanism leading to this increase in variability, a mechanism that ended up unlocking the possibility of more complex life forms such as all subsequent multicellular organisms.

## Materials and Methods

### Gene Annotations.

Gene lengths were obtained from the General Transfer Format gene annotation files corresponding to the 33,627 genomes downloaded from the different specialized web servers from Ensemblgenomes (release 49) and Ensembl (release 98) (12). Specifically, the total number of genomes obtained from each web server corresponds to 31,943 prokaryotes from EnsemblBacteria (1,229 Archaea and 30,714 Bacteria), 237 protists from EnsemblProtists, 96 plants from EnsemblPlants, 1,014 Fungi from EnsemblFungi, 115 invertebrates (Metazoa but not Vertebrata) from EnsemblMetazoa, and 222 vertebrates (Vertebrata) from Ensembl. Although protists, plants, and invertebrates are not monophyletic groups, they are already well categorized by Ensembl.

### Protein Annotations.

Each protein length has been directly computed from its corresponding protein sequence. A total of 9,913 different reference proteomes were downloaded from Uniprot (31) (release 2021-02): 330 for Archaea, 7,997 for Bacteria, and 1,586 for Eukaryota. The categorization is well done already by Uniprot. Nevertheless, a finer subcategorization of Eukaryota coherent with that of Ensembl was carried out using the taxonomic hierarchical classification provided by Uniprot. This resulted in 156 protists, 184 plants, 772 Fungi, 226 invertebrates, and 248 vertebrates.

### Statistical Analyses to Fit Gene and Protein Length Distributions.

To determine that the length distributions of both genes and proteins are lognormal all over the tree of life, we have fitted gene and protein distributions to the main competing right-skewed distributions: lognormal, gamma, Weibull, exponential, log logistic, and Gumbel distributions. In *SI Appendix*, Fig. S2, we show histograms of log-likelihood differences between the best fit with a lognormal distribution and the best fit with the other distributions using the Python library *Reliability*. The gene length distribution for a species is fitted to these six distributions obtaining as an outcome the log-likelihood for each fit. The distribution function that best fits that particular gene length distribution will be the one with the highest log-likelihood. For each gene length distribution, we calculated the difference between the log-likelihood for the lognormal and the competing distribution, and plotted the histogram of the differences (*SI Appendix*, Fig. S2, *Left*). The same procedure was applied for the protein length distributions (*SI Appendix*, Fig. S2, *Right*).

### Gene Length Growth Through Time.

To validate the exponential and linear growth of the mean gene length and mean gene log length, respectively, with real data, we used Timetree of Life v5 (Timetree2022). Timetree provides an estimate of the divergence time between two given lineages. Using *H. sapiens* as a reference, we assigned the divergence times to each phyletic group. These values represent a lower limit for the age of appearance of a lineage. For instance, the estimated divergence time for Aves indicates that they diverged from modern humans approximately 319 My ago, even though Aves appeared later. Additionally, divergence times with *H. sapiens* may vary among species inside the same group, particularly for Primates and nonprimate Mammals. Thus, we used the average divergence time for all the organisms within each of these two groups included in our dataset.

To attribute a representative mean gene length $\langle L\rangle$ and mean gene log length $\langle \log L\rangle$ to each time *t*, we estimated them by averaging these values from all species within each group, only considering groups with a minimum of 20 species and the same divergence time. We were able to provide the taxonomic classification to the majority of the genome annotations from Ensembl (33,459 out of the total of 33,627). In order to do that, we associated the genome annotations from Ensembl to the taxonomic classifications of almost 2.7 Million species downloaded from Uniprot on 19.11.2021. Taxons with at least 20 species were selected, resulting in 30,555 Bacteria, 1,228 Archaea, 93 Viridiplantae, 991 Fungi, 86 Arthropoda, 60 Actinopterygii, 24 Aves, 96 nonprimate Mammalia, and 26 Primates. After the taxonomic classification, 237 Eukaryota remained, all of them being protists.

This approach aims to mitigate fluctuations arising from particularities in the evolutionary history of individual species. We computed the averaged mean gene length (and mean gene log length) for each group by averaging the individual mean gene length values (and mean gene log length values) for all species within the group and assigning these averages to the entire group. It is important to note that, due to the growth tendency of genes, this approach will now yield an upper limit for the mean gene length present at the time of the first appearance of representatives within the group.

### Merging Ensembl and Uniprot Annotations.

Ensembl and Uniprot annotations have been associated through the taxonomic identifier provided independently by both repositories. As a result, we obtained 7,669 species with information on both repositories. These species are distributed within the following major groups as follows: Archaea (283), Bacteria (6,459), protists (114), plants (72), Fungi (566), invertebrates (63), and vertebrates (112).

Those 7,669 species have been filtered considering only the species for which $3\cdot \left\langle L_{p}\right\rangle \leq \left\langle L\right\rangle$, where $\left\langle L_{p}\right\rangle$ and ⟨L⟩ indicate mean protein and gene length, respectively. This is intended to clean the dataset by ensuring that the lengths of sequences that code for protein are not longer than their corresponding protein-coding genes.

This first filtering resulted in a total of 6,705 species. To make sure we had a minimum number of genes per species to avoid large fluctuations, from the latter list of species, we discarded those instances when the total number of genes or proteins was lower than 500. This resulted in 6,683 species. Finally, we filtered out species for which Ensembl and Uniprot differ by more than 5% in their corresponding number of proteins and genes, resulting in a total of 6,519 genomes. This led to the final sample of major groups used in Fig. 3: Archaea (227), Bacteria (5,468), protists (91), plants (59), Fungi (533), invertebrates (49), and vertebrates (92). In any case, the same qualitative results are obtained when using the total of 7,669 species without filtering.

### Identifying the Threshold Value.

The threshold shown in Fig. 3 corresponds to a mean gene length around 1,500 base pairs. However, the specific value may not seem clear due to the dispersion of the cloud of points. As a consequence, we have plotted an equivalent graph but considering only the best annotated genomes. The genome assembly identifier and its corresponding assembly status was associated to the taxonomy id of the species. 23,098 Eukarya and 409,259 Prokarya genome assembly reports were downloaded from the National Center for Biotechnology Information (genomes) on 22.6.2022. Then we filtered again the merged set of 6,519 retrieving only those with the best genome assembly status (“Complete genome” or “Chromosome”). Using only this selection of the best genomes, we obtain *SI Appendix*, Fig. S9, *Bottom*, where one can better appreciate how the change of regime happens at mean gene length ≈1,500 base pairs.

### Averaging Data for Groups.

To check whether mean gene length correlates better with organismal complexity than mean protein length as shown in *SI Appendix*, Fig. S6, we grouped species using the taxonomy as above and averaged the mean gene lengths in each group. That is, in order to minimize the influence of the variance of these values and the statistical particularities that a certain organism could have, we kept only those groups that had at least 20 representatives in both datasets.

For the case of genes, this led to 1,228 Archaea, 30,555 Bacteria, 237 protists, 93 Viridiplantae, 991 Fungi, 86 Arthropoda, 60 Actinopterygii, 24 Aves, 96 nonprimate Mammalia, and 26 Primates. For the case of proteins, this procedure led to 330 Archaea, 7,986 Bacteria, 154 protists, 178 Viridiplantae, 726 Fungi, 115 Arthropoda, 65 Actinopterygii, 60 Aves, 75 nonprimate Mammalia, and 24 Primates.

### Growth of Coding and Noncoding Intragenic Sequences.

We can decompose the length of each gene i=1,2,... as

$$L_{i}\left(t\right)=l_{i}\left(t\right)+{\overset{¯}{l}}_{i}\left(t\right),$$

where $l_{i}\left(t\right)$ and ${\overset{¯}{l}}_{i}\left(t\right)$ represent the length of coding and noncoding sequences, respectively, in that gene. Thus,

$$\langle L(t)\rangle =\langle l(t)\rangle +\langle \overset{¯}{l}(t)\rangle ,$$

and the fraction of noncoding sequences in the genome is

[8] $$\begin{array}{c} \rho \left(t\right)=\frac{\left\langle \overset{¯}{l}(t)\right\rangle }{\left\langle L(t)\right\rangle }=1-\frac{\left\langle l(t)\right\rangle }{\left\langle L(t)\right\rangle }. \end{array}$$

If we assume that for mean gene lengths ⟨L(t)⟩≤1,500 base pairs, genes grow incorporating exclusively coding sequences ($\langle \overset{¯}{l}(t)\rangle =0$), then in this regime ⟨L(t)⟩=⟨l(t)⟩.

Then, upon reaching the threshold of 1,500, the variance of the distribution will be determined by ⟨L(t)⟩=1,500, given by Taylor’s law. If from that moment on, genes grow incorporating exclusively noncoding sequences, the lengths of the coding sequences *l*_*i*_ will not vary and will keep the former distribution. Now, a distribution of noncoding sequences will grow on it. Note that *l*_*i*_ is a lognormal distribution, and $L_{i}\left(t\right)$ grows following also a lognormal distribution, ${\overset{¯}{l}}_{i}\left(t\right)$ being the distribution coming from the difference of the other two. But since the sum and difference of two correlated lognormal random variables is approximately lognormal (5), then we will get approximately a lognormal distribution for the lengths of noncoding sequences.

We conducted simulations to model this growth process of coding and noncoding intragenic sequences using as the initial conditions a typical bacterium’s gene length distribution. Initially, only coding sequences were incorporated until the simulation reached a mean length of 1,500 base pairs. At this point, we saved a backup of the gene lengths. The simulation then continued, now incorporating exclusively noncoding sequences until the mean gene length matched that of modern humans, specifically $\langle L_{\text{humans}}\rangle =68,287$ base pairs.

Upon completion of the simulation, for each gene, we determined the difference between its final length and the length at the time of the backup. The resulting discrepancy represents the noncoding sequence length, while the length at the backup corresponds to the coding sequence length. The final distributions of log(lengths) for both the coding and noncoding portions of each simulated gene are depicted in *SI Appendix*, Fig. S8, *Top*.

Next, we compare the observed distribution for *H. sapiens* with the simulated one, considering both coding and noncoding genes. To facilitate this comparison, we paired each human protein in Uniprot with its corresponding gene in Ensembl. While some genes can express multiple proteins through alternative splicing, the reference proteome provides the canonical protein for each gene. The coding-sequence length for each gene is estimated as three times the protein length, and the noncoding-sequence length is determined as the difference between the total gene length and three times the protein length. Notably, the observed distributions for *H. sapiens* closely resemble our simulations (*SI Appendix*, Fig. S8, *Bottom*), suggesting that our simple multiplicative growth model captures the fundamental aspects of the evolutionary process.

### Measuring the Increase of States Around the Critical Point.

A property of lognormal distributions is that they are determined by their mean and variance. Also, according to Taylor’s law, the variance is determined by the mean (Eqs. **3** and **4**). As a consequence of these two facts, mean gene length by itself completely determines the gene length distribution. However, this refers to total gene length regardless of whether it corresponds to a coding or noncoding sequence. Indeed, as noted in *SI Appendix*, Fig. S8, the gene length histograms for *H. sapiens* are significantly different for the two types of sequences. The fraction of noncoding sequences (*ρ*) is quite variable. Thus, we define a species by the pair (⟨L⟩,ρ). To categorize states, we logarithmically binned the horizontal axis for mean gene length and linearly binned the vertical axis for *ρ* (ranging from 0 to 1) into 100 uniformly distributed bins. This binning creates a grid where each square represents a distinct state. A square is assigned a value of 1 if at least one species with a specific pair value (⟨L⟩,ρ) falls within it, and 0 otherwise. Subsequently, for a given mean gene length bin, the fraction of states is determined by summing the values in its column and dividing those by 100. A higher fraction of states indicates that different species with the same mean gene length exhibit contrasting *ρ* values. This measure, as illustrated by the *Inset* in Fig. 4 (white squares), peaks around $L_{c}=1,500$ base pairs, aligning with expectations for a tipping point.

It can be argued that the higher dispersion of values around the tipping point could result from an artifact as we have more organisms around this point. To discard this possibility, we have performed a bootstrap. For each mean gene length bin, we have randomly chosen a subsample of up to 10 organisms (in those bins where there were less than 10, we kept all of them) and calculated the (⟨L⟩,ρ) states for those subsets. After this procedure, the peak remains. This is also the case for the entropy of *ρ* values in Fig. 4, which also shows a maximum around the same location (*SI Appendix*, Fig. S10).

## Supplementary Material

Appendix 01 (PDF)

## Data Availability

Data and code have been deposited in GitHub (https://github.com/emuro/gene_length). Previously published data were used for this work [Ensembl (https://www.ensembl.org/) and Uniprot (https://www.uniprot.org/)].
