# Supplementary material for: The emergence of eukaryotes as an evolutionary algorithmic phase transition
Source: Proc Natl Acad Sci U S A. 2025 Mar 27;122(13):e2422968122. doi: 10.1073/pnas.2422968122 (PMC12002324; doi:10.1073/pnas.2422968122)
Supplement: Supplementary file 1 — Appendix 01 (PDF) [file pnas.2422968122.sapp.pdf]

Supporting Information for

The emergence of eukaryotes as an evolutionary algorithmic phase transition

Enrique M. Muro, Fernando J. Ballesteros, Bartolo Luque, and Jordi Bascompte

Corresponding Author: Jordi Bascompte.
E-mail: jordi.bascompte@uzh.ch

This PDF file includes:

Supporting text
Figs. S1 to S10
SI References

Contents

1 Multiplicative Stochastic Model 3

2 Lognormal distributions 3

3 Exponential growth of mean gene length $\langle L(t) \rangle$ over time 3

4 Linear growth of mean gene log length $\langle \log L(t) \rangle$ over time 4

5 Relationship between mean length and logarithmic mean length 5

6 Generalised Taylor's Law and asymptotic Taylor's Law 5

7 Length distribution and Taylor's law for proteins 6

8 Growth laws for proteins 7

9 Signal of critical slowing down in Fungi 7

10 Relationship between the phase transition and Taylor's law 7

11 Algorithmic transition and easy-hard-easy pattern 8

12 Figures 10

13 SI References 20

Supporting Information Text

1. Multiplicative Stochastic Model

Given that the observed length distributions of protein-coding genes (genes hereafter) are lognormal and that lognormal distributions commonly arise from various multiplicative processes, we start by considering a simple multiplicative stochastic model of gene growth. The process is as follows:

1. We initialise N genes with initial lengths $L_i(0)$, where $i = 1, 2, \ldots, N$.
2. At each iteration of the algorithm, denoted as $s = 1, 2, \ldots$, one gene k is randomly selected, and its length $L_k(s-1)$ is multiplied by a random number $\zeta \in [z_1, z_2]$ generated from a probability density function $f(\zeta)$. The lengths of the remaining $N-1$ genes remain unchanged: $L_i(s) = L_i(s-1)$, $i \neq k$.

2. Lognormal distributions

At each step in our algorithm we choose a given gene at random rather than all at once. Therefore, the model is asynchronous. But, as in a genome of N genes, each gene will be multiplied by a random value ζ on average once every N steps of the algorithm, we can define the time in our model as $t \equiv s/N = 1, 2, \ldots$

The length $L(t)$ of any gene behaves like a random variable that evolves according to the stochastic equation:

$$L(t) = \zeta_t \cdot L(t-1) = L(0) \cdot \prod_{n=1}^{t} \zeta_n,$$

where the multiplicative factors ζ_n are identically-distributed random variables with probability density function $f(\zeta)$. Taking logarithms on both sides, we have:

$$\log L(t) = \log L(0) + \sum_{n=1}^{t} \log \zeta_n.$$

If the probability density of the random variable $\log \zeta$ has first and second finite moments, the Central Limit Theorem assures us that, for $t \gg 1$, the random variable $\log L(t)$ will be normally distributed. Thus, $L(t)$ will follow a lognormal distribution:

$$f\left(L(t)\right) = \frac{1}{L(t)\varsigma(t)\sqrt{2\pi}} \exp\left(-\frac{\left(\log L(t) - \mu(t)\right)^2}{2\varsigma^2(t)}\right),$$

where $\mu(t)$ and $\varsigma^2(t)$ are the expected value and variance, respectively, of the random variable $\log L(t)$; that is: $\mu(t) = \langle \log L(t) \rangle$ and $\varsigma^2(t) = \mathrm{Var}\left(\log L(t)\right)$.

3. Exponential growth of mean gene length $\langle L(t) \rangle$ over time

Let's see that the model predicts an exponential growth of the mean length of genes. Consider that we have $i = 1, 2, ..., N$ genes and $m = 1, 2, ..., M$ replicates of the multiplicative process. If we indicate $L_i(s, m)$ as the length of gene i at iteration s and replicate m, the following set of stochastic equations holds:

$$\begin{aligned} L_i(s,m) &= \frac{1}{N} \cdot \zeta_{i,m} \cdot \ L_i(s-1,m) + \left(1 - \frac{1}{N}\right) \cdot 1 \cdot \ L_i(s-1,m) \\ &= \left(1 + \frac{\zeta_{i,m} - 1}{N}\right) \cdot \ L_i(s-1,m), \end{aligned}$$

where each equation describes that at each iteration s, the length of gene i at replicate m, $L_i(s, m)$, will:

- be multiplied by a stochastic multiplicative factor $\zeta_{i,m}$ drawn from the probability density function $f(\zeta)$ with probability $1/N$, or
- remain unchanged, i.e., be multiplied by 1, with probability $1 - 1/N$.

Next, let's denote by $\langle \zeta \rangle$ the average value of $f(\zeta)$, that is:

$$\langle \zeta \rangle = \int_{z_1}^{z_2} \zeta \cdot f(\zeta) d\zeta.$$

If we now average across the M replicates, and keeping in mind that random variables length and multiplicative factor are independent, we obtain:

$$\langle L_i(s) \rangle = \frac{1}{M} \sum_{m=1}^{M} L_i(s,m) = \frac{1}{M} \sum_{m=1}^{M} \left(1 + \frac{\zeta_{i,m} - 1}{N}\right) \cdot \ L_i(s-1,m)$$

$$= \left(1 + \frac{\langle\zeta\rangle - 1}{N}\right) \cdot \ \langle L_i(s-1)\rangle \,.$$

So, the length of an arbitrary gene i after s steps will be on average:

$$\langle L_i(s)\rangle = L_i(0) \cdot \left(1 + \frac{\langle\zeta\rangle - 1}{N}\right)^s .$$

Adding the N equations for all genes $i = 1, \ldots, N$ and dividing by N, we find the growth equation for the mean gene length, $\langle L(s)\rangle$, for $N \gg 1$:

$$\langle L(s)\rangle = L_0 \cdot \left(1 + \frac{\langle\zeta\rangle - 1}{N}\right)^s \approx L_0 \cdot \exp\left((\langle\zeta\rangle - 1) \cdot \frac{s}{N}\right),$$

where L_0 is the mean value of the initial lengths:

$$L_0 = \frac{1}{N}\sum_{i=1}^{N} L_i(0).$$

As $t = s/N$, the model predicts an exponential growth of mean gene length over time:

$$\langle L(t)\rangle = L_0 \cdot \exp\left((\langle\zeta\rangle - 1) \cdot t\right). \qquad [1]$$

Eq. (1) only depends on the mean value of the distribution of ζ, but not on its specific probability density function $f(\zeta)$. This result has been confirmed through simulation across various distributions $f(\zeta)$ and values of L_0. It is noteworthy that the simulations exhibit considerable dependency on the history of each realisation and the specifics of the multiplicative factors generated therein. Generally, for a given simulation, the mean gene length follows a geometric random walk. It is through averaging across multiple realisations that the exponential growth characteristic holds true.

Also, it is important to note that the predicted exponential growth of mean gene length is robust even when additive processes are also incorporated. Indeed, the presence of a multiplicative growth process is sufficient, as when it competes with several additive processes, the geometric growth of the former rapidly dominates over the arithmetic growth of the latter, essentially nullifying their effect. Asymptotically, we can disregard the impact of additive growth processes.

Fig. S3 (top) illustrates a clear exponential growth ($R^2 = 0.79$), consistent with the prediction in Eq. (1) with $L_0 = 542$ and $\langle\zeta\rangle = 1.0011$. However, it is crucial to approach these estimates with caution. Note that $L_0 = 542$ serves as a preliminary estimate for the mean gene length of the Last Universal Common Ancestor (LUCA).

4. Linear growth of mean gene log length $\langle \log L(t)\rangle$ over time

Note that, in general for an arbitrary distribution, $\langle \log L(t)\rangle$ is not equal to $\log \langle L(t)\rangle$. Let us deduce the growth equation for $\langle \log L(t)\rangle$ according to our model.

Because, as noted above, $L_i(s)$ is as a random variable, for every gene $i = 1, \ldots, N$ we can write the following stochastic equation:

$$L_i(t) = \ L_i(0) \cdot \prod_{n=1}^{t} \zeta_n .$$

Now taking logarithms on both sides:

$$\log L_i(t) = \log L_i(0) + \sum_{n=1}^{t} \log \zeta_n = \log L_i(0) + \langle \log \zeta\rangle \cdot t,$$

where:

$$\langle \log \zeta\rangle = \int_{z_1}^{z_2} \log \zeta \cdot f(\zeta) d\zeta.$$

Adding the equations for the N genes and dividing by N, we find that the logarithmic mean length increases linearly:

$$\langle \log L(t)\rangle = G_0 + \langle \log \zeta\rangle \cdot t, \qquad [2]$$

where G_0 is the logarithmic mean of the initial conditions:

$$G_0 = \frac{1}{N}\sum_{i=1}^{N} \log L_i(0).$$

As it was the case for Eq. (1), Eq. (2) only depends on the logarithmic mean of the distribution of ζ, but not on its specific probability density function $f(\zeta)$. We have confirmed this result through simulations for various probability distributions $f(\zeta)$ and values of G_0.

Fig. S3 (bottom) shows the fit of the data, with a goodness of fit of $R^2 = 0.79$, compatible with the linear relationship at Eq. (2) with $G_0 = 6.20$ and $\langle \log \zeta\rangle = 0.0008$ (again estimates that we must take with caution). Note that $G_0 = 6.20$ is an estimate of the mean log length of LUCA's genes.

5. Relationship between mean length and logarithmic mean length

Note that, irrespective of the model used, the fit of data described above suggests that both the mean and the logarithmic mean of the lengths of protein-coding genes grow exponentially and linearly, respectively, in accordance with Eq. (1) and Eq. (2). Once it's established that these two growth patterns exist, it inevitably implies a relationship between $\log\langle L(t)\rangle$ and $\langle \log L(t)\rangle$.

From Eq. (1) and Eq. (2), eliminating time, we can find the following linear relationship:

$$\log \langle L(t)\rangle = \left(\frac{\langle\zeta\rangle - 1}{\langle \log \zeta\rangle}\right)\cdot \langle \log L(t)\rangle + \left(\log L_0 - \frac{(\langle\zeta\rangle - 1)\cdot G_0}{\langle \log\zeta\rangle}\right). \quad [3]$$

Substituting the estimated values into Eq. (1) and Eq. (2), we obtain the following prediction: $\log \langle L(t)\rangle = 1.37 \cdot \langle \log L(t)\rangle - 2.23$. However, the fit of the data in Fig. S4 (red line), which is notably strong ($R^2 = 0.97$), is: $\log \langle L(t)\rangle = 1.20\cdot \langle \log L(t)\rangle - 1.11$. The discrepancy can be attributed to the uncertainty associated with the data in the graphs for the growth Eq. (1) and Eq. (2). We will consider the latter fit as more reliable for estimating parameters. Our further analytical work will allow us to find a better fit to the predicted relationship between $\log \langle L(t)\rangle$ and $\langle \log L(t)\rangle$ (see Eq. (13) and following text).

6. Generalised Taylor's Law and asymptotic Taylor's Law

Without the need to appeal to a specific growth model, the data support that gene length distributions are lognormal, and that the mean and logarithmic mean grow according to Eq. (1) and Eq. (2). We will now see that, when these three statements are true, the inevitable consequence is that the variance is determined by the mean. More specifically, the consequence is a generalised Taylor's law that relates the mean and the second raw moment of the distributions through a scaling law (1).

For any random variable $L(t)$ following a lognormal distribution, the following relationships hold regarding its mean $\langle L(t)\rangle$ and variance $\sigma^2(t)$:

$$\langle L(t)\rangle = \exp\left(\mu(t) + \frac{\varsigma^2(t)}{2}\right) = \exp\left(\langle \log L(t)\rangle + \frac{\varsigma^2(t)}{2}\right), \quad [4]$$

$$\begin{aligned}\sigma^2(t) &= \exp\left(2\mu(t) + \varsigma^2(t)\right)\left[\exp \varsigma^2(t) - 1\right] \\ &= \exp\left(2\langle \log L(t)\rangle + 2\varsigma^2(t)\right) - \exp\left(2\langle \log L(t)\rangle + \varsigma^2(t)\right). \end{aligned} \quad [5]$$

From Eq. (4) we obtain:

$$\varsigma^2(t) = 2\log\langle L(t)\rangle - 2\langle \log L(t)\rangle,$$

which substituting into Eq. (5) allows us to express the variance of the length of genes as a function of their mean length and their mean logarithmic length:

$$\sigma^2(t) = \frac{\langle L(t)\rangle^4}{\left(\exp\langle \log L(t)\rangle\right)^2} - \langle L(t)\rangle^2. \quad [6]$$

Eq. (6) holds for any lognormal distribution, but in our case it happens that $\langle \log L(t)\rangle$ grows linearly with time according to Eq. (2). So that:

$$\sigma^2(t) = \frac{\langle L(t)\rangle^4}{\exp\left(2G_0\right)\cdot\left(\exp\left(\langle\log\zeta\rangle\cdot t\right)\right)^2} - \langle L(t)\rangle^2.$$

In our case, the mean gene length grows exponentially with time according to Eq. (1), so we can write:

$$\sigma^2(t) = \frac{\langle L(t)\rangle^4}{\exp\left(2G_0\right)\cdot\left(\exp\left(\frac{(\langle\zeta\rangle-1)\langle\log\zeta\rangle}{(\langle\zeta\rangle-1)}\cdot t\right)\right)^2} - \langle L(t)\rangle^2$$

$$\sigma^2(t) = \frac{L_0^{\frac{2\langle\log\zeta\rangle}{\langle\zeta\rangle-1}}}{\exp\left(2G_0\right)}\cdot\langle L(t)\rangle^{4-\frac{2\langle\log\zeta\rangle}{\langle\zeta\rangle-1}} - \langle L(t)\rangle^2, \quad [7]$$

which we can rewrite as:

$$\sigma^2(t) = a\cdot\langle L(t)\rangle^{\beta} - \langle L(t)\rangle^2, \quad [8]$$

where we can see that the exponent β only depends on the mean and the logarithmic mean of the multiplicative factor, but not on its specific probability density function $f(\zeta)$:

$$\beta = 4 - \frac{2\langle\log\zeta\rangle}{\langle\zeta\rangle - 1}, \quad [9]$$

and the coefficient a depends on the exponent β and the initial conditions:

$$a = \frac{L_0{}^{4-\beta}}{\exp\left(2G_0\right)}. \quad [10]$$

Here, we observe the advantage of utilising the mean value of gene length, as Eq. (8) indicates that the mean length of protein-coding genes fully determines the variance, i.e., the lognormal distribution of their lengths.

Eq. (8), for values $\langle L(t)\rangle \gg 1$ and $\beta > 2$ (as is the case), behaves asymptotically like a standard Taylor's law (Fig. 2):

$$\sigma^2(t) = a \cdot \langle L(t)\rangle^{\beta} \left(1 - \frac{1}{a \cdot \langle L(t)\rangle^{\beta-2}}\right) \approx a \cdot \langle L(t)\rangle^{\beta}. \qquad [11]$$

As a consequence of the growth equations (Eq. (1) and Eq. (2)), the phyletic groups should appear according to their evolutionary order. Therefore, the Taylor's law should inherit this temporal sequence, a notion supported by the data (see Fig. 2). Thus, since a greater mean gene length implies a later appearance in the history of life, mean gene length serves as a proxy for the complexity of an organism (Fig. S6, left).

To correctly estimate a and β through this asymptotic relationship, we should limit to species with $\langle L(t)\rangle \gg 1$, as is the case of Vertebrata (red points in Fig. 2). If we fit the data from Vertebrata, we get: $\sigma^2(t) \approx 0.19 \cdot \langle L(t)\rangle^{2.28}$, with a goodness of fit $R^2 = 0.95$.

Note that, if we take all the data including low values of $\langle L(t)\rangle$, due to the fact that the approximation used in Eq. (11) is valid only for $\langle L(t)\rangle \gg 1$, we would be overestimating the exponent β and underestimating the coefficient a, in addition to obtaining worse goodness of fit, as it happens: $\beta = 2.51$, $a = 0.016$ and $R^2 = 0.92$ (see Fig. 2 and Fig. S5, left).

As the variance of a distribution can be calculated from its first and second raw moment:

$$\sigma^2(t) = \langle L^2(t)\rangle - \langle L(t)\rangle^2,$$

Eq. (8) turns out to be an exact scaling law for $\langle L^2(t)\rangle$, i.e. a generalised Taylor's law (1) for the second raw moment of the length:

$$\langle L^2(t)\rangle = a \cdot \langle L(t)\rangle^{\beta}. \qquad [12]$$

Using Eq. (12) we do not have to limit ourselves to $\langle L(t)\rangle \gg 1$, but we can use the entire data set. Fitting all the data to Eq. (12) yields a better goodness of fit: $R^2 = 0.98$, with $a = 0.21$ and $\beta = 2.29$ (Fig. S5, right). The latter values are practically identical to the asymptotic ones formerly found using Eq. (11) and the Vertebrata data. We will take these estimates as better due to the quality and quantity of the data used, in addition to the better goodness of fit.

Note that Eq. (9) and Eq. (10) allow us to rewrite Eq. (3) as:

$$\log\langle L(t)\rangle = \frac{2}{4-\beta}\langle \log L(t)\rangle + \frac{\log a}{4-\beta}. \qquad [13]$$

The last estimated values allow us to obtain the following prediction for the parameters of Eq. (13): $\log\langle L(t)\rangle = 1.17 \cdot \langle \log L(t)\rangle - 0.91$. These values are practically identical to the ones obtained fitting these data.

The data fits of the exponential and linear growth curves gave us estimates for G_0, L_0, $\langle\zeta\rangle$ and $\langle\log\zeta\rangle$. The fit of data to Taylor's for the second raw moment gave us estimates for a and β. Both groups of estimates come from two different data sets, and yet the model states that both must be related through Eq. (9) and Eq. (10). More specifically, the values of a and β determine the relationship between $\langle\zeta\rangle$ and $\langle\log\zeta\rangle$, and between G_0 and L_0.

Let us observe that if we take the estimation $G_0 = 6.20$ from Eq. (10), we obtain: $L_0 = 566$. If we instead opt for the estimation $L_0 = 542$, using the same equation, we obtain $G_0 = 6.16$. By averaging these estimations, we have $G_0 = (6.20 + 6.16)/2 = 6.18$ and $L_0 = (566 + 542)/2 = 554$. Now, if we repeat the same operation with these new values using Eq. (10), we find surprisingly no variation.

Now, taking the initial estimations $\langle\zeta\rangle = 1.0011$ and $\langle\log\zeta\rangle = 0.0008$ in Eq. (9), we obtain $\langle\zeta\rangle = 1.00093$ and $\langle\log\zeta\rangle = 0.00094$. Averaging these, we get: $\langle\zeta\rangle = (1.0011 + 1.00093)/2 = 1.00101$ and $\langle\log\zeta\rangle = (0.0008 + 0.00094)/2 = 0.00087$, which again stabilise under the same operation.

This leads us to our final estimations: $G_0 = 6.18$, $L_0 = 554$, $\langle\zeta\rangle = 1.00101$, and $\langle\log\zeta\rangle = 0.00087$, along with $a = 0.21$ and $\beta = 2.29$, satisfy Eq. (9) and Eq. (10). Finally, substituting these values into Eq. (3), we obtain $\log\langle L(t)\rangle = 1.16 \cdot \langle\log L(t)\rangle - 0.86$, a result close to the fit of the data (see Fig. S4, black line), thereby confirming the coherence of the model.

7. Length distribution and Taylor's law for proteins

For prokaryotes and organisms without non-coding sequences (nCDS), the length of a given protein (measured in amino acids) is directly 1/3 the length of the corresponding gene (measured in base pairs). As we have seen, this regime mainly happens at mean gene lengths under 1,500 base pairs (mean protein lengths under 500 amino acids). We find that for these organisms, the distributions of genes and proteins coincide, once scaled by this factor 3 (see Fig. S7, left). Therefore, once scaled, for $\langle L(t)\rangle \leq 1,500$ and using Eq. (12), we find:

$$\sigma_p^2(t) = 3^{\beta-2} a \langle L_p(t)\rangle^{\beta} - \langle L_p(t)\rangle^2,$$

and a generalized Taylor's law for proteins:

$$\langle L_p^2(t)\rangle = 3^{\beta-2} a \cdot \langle L_p(t)\rangle^{\beta}.$$

Substituting the estimates $a = 0.21$ and $\beta = 2.29$ previously obtained, we get:

$$\sigma_p^2(t) = 0.29 \cdot \langle L_p(t)\rangle^{2.29} - \langle L_p(t)\rangle^2,$$

and

$$\langle L_p^2(t) \rangle = 0.29 \cdot \langle L_p(t) \rangle^{2.29}.$$

In Fig. S5 (inset, right) we fit these values for proteins, obtaining $\langle L_p^2(t)\rangle = 0.39 \cdot \langle L_p(t)\rangle^{2.24}$, values which are close to the theoretical ones. But note that the fit includes all genomes, not only those with $\langle L(t)\rangle \leq 1,500$. And yet it works. As we said, beyond a mean gene length of $1,500$ base pairs, mean protein length is stabilised around 500 amino acids, but all these organisms fall over the same region of the scaling law because their associated variances are also similarly stabilised. This implies that protein length distributions for organisms with mean gene length beyond $1,500$ should be very similar, as confirmed by our data and by ref. 2.

Summing up:

1. If the mean length of a species' genes $\langle L(t)\rangle$ is less than or equal to $1,500$ base pairs, the length distribution of its proteins will be close to a lognormal with mean $\langle L_p(t)\rangle = \langle L(t)\rangle/3$ and variance $\sigma_p^2(t) = \sigma^2(t)/9$.

2. If the mean length of a species' genes $\langle L(t)\rangle$ is greater than $1,500$ base pairs, then the protein length distribution will be close to lognormal $\left(500,\ 1.8 \times 10^5\right)$.

8. Growth laws for proteins

A direct consequence of the data is that as long as $\langle L(t)\rangle \leq 1,500$ base pairs, proteins grew along evolution hand to hand with the growth of genes. As a consequence, using our estimations of $G_0 = 6.18$, $L_0 = 554$, $\langle \zeta \rangle = 1.00101$, and $\langle \log \zeta \rangle = 0.00087$, the scaling between gene and protein lengths determines the two growth laws of proteomes for organisms with $\langle L(t)\rangle \leq 1,500$:

$$\langle L_p(t)\rangle = \frac{\langle L(t)\rangle}{3} = \frac{L_0}{3} \exp\left(\left(\langle \zeta \rangle - 1\right) \cdot t\right) = 184.6 \cdot \exp\left(0.00101 \cdot t\right),$$

$$\langle \log L_p(t)\rangle = \langle \log\left(L(t)/3\right)\rangle = G_0 - \log 3 + \langle \log \zeta \rangle \cdot t = 5.081 + 0.00087 \cdot t,$$

which gives us an estimate of the mean and mean logarithmic length of the LUCA proteome: $\langle L_p(0)\rangle = 184.6$ and $\langle \log L_p(t)\rangle = 5.081$. For organisms with $\langle L(t)\rangle \geq 1,500$ it holds: $\langle L_p(t)\rangle \approx 500$, and $\langle \log L_p(t)\rangle \approx 5.9$, i.e., these means are approximately constant.

Therefore, in Taylor's law for proteins, the evolutionary order of appearance should only be inherited from the mean growth equations (Eq. (1) and Eq. (2)) for $\langle L(t)\rangle \leq 1,500$. This order is lost for mean protein lengths larger than 500 amino acids because the distribution of their lengths is practically not altered. While $\langle L(t)\rangle$ is a good proxy for complexity, $\langle L_p(t)\rangle$ only works below the threshold (Fig. S6, right).

9. Signal of critical slowing down in Fungi

As noted above, fungi are along with prokaryotes the group that dominates around the critical point L_c. Fungi are also the largest group in our data set after Bacteria, so we can be confident of having sufficient completeness of data. As shown in Fig. S9 (top), there is a marked peak in the histogram at $L_c = 1,500$. This indicates an overabundance of fungal species with that same mean gene length. That is, it hints towards an increase in the number of states around the critical value. As discussed in main text, this is a sign of critical slowing down characteristic of second-order phase transitions. Also, the specific location of the peak in fungi constitutes an additional quantification of the critical point in Figs. 3 and 4. This complements our exploration of the robustness of our estimation of the critical point at $L_c \approx$ 1,500 base pairs by limiting our analysis to the best annotated genomes (see Materials and Methods).

Overall, therefore, our combined results indicate that the behaviour observed in Figs. 3 and 4 is not due to a threshold phenomenon, but to a second-order phase transition. In the following sections we will study which kind of phase transition it is.

10. Relationship between the phase transition and Taylor's law

Since we are dealing with lognormal distributions, we can express the mean as a function of the median. Therefore, we can use the median instead of the mean as the control parameter.

For example, Eq. (6) expresses the variance of genes as a function of the mean and logarithmic mean of the gene lengths. If we combine it with Eq. (8), we have:

$$\frac{\langle L(t)\rangle^4}{\left(\exp\langle \log L(t)\rangle\right)^2} = a \cdot \langle L(t)\rangle^{\beta}.$$

Since the median $M(t)$ of a lognormal fulfills the following:

$$M(t) = \exp\langle \log L(t)\rangle,$$

we obtain a scaling law between the mean and the median of the lengths:

$$\langle L(t)\rangle = a^{\frac{1}{4-\beta}} M^{\frac{2}{4-\beta}}(t).$$

Using the median of the lengths as a control parameter in the transition equation (Eq. 4 in main text), we have:

$$\rho(M) = \begin{cases} 0 & \text{if } M \leq M_c \\ 1 - \left(\frac{M_c}{M}\right)^{\frac{2}{4-\beta}} & \text{if } M > M_c \end{cases}$$

where:

$$M_c = \frac{L_c^{\frac{4-\beta}{2}}}{\sqrt{a}}.$$

The previous transition shows an explicit dependence with the exponent β and coefficient a of Taylor's law, which relates the two main results of our paper. This is not the case, however, when mean gene length—instead of the median of gene length—is used as the control parameter. But this makes no sense since, as noted above, the mean can be expressed as a function of the median. This simple change of variables should maintain the relationship between Taylor's law and the phase transition. Indeed, we show this is the case in the following section, where we uncover this dependence hiding in the value of the critical mean gene length L_c. Importantly, we will do so by assuming that the phase transition belongs to the class of algorithmic transitions, an assumption that will also allow us to predict the critical point.

11. Algorithmic transition and easy-hard-easy pattern

If we define a reduced mean gene length $r \equiv (\langle L \rangle - L_c)/L_c$ from equation (4) in main text, then the order parameter $\rho(r)$ (the fraction of gene non-coding sequences) above and close to the critical point L_c follows the scaling law: $\rho(r) \sim r^{\alpha}$, with a critical exponent $\alpha = 1$ similar to a classical percolation transition in a network (3). As noted in refs. 4 and 5 and discussed in main text, phase transitions like the former can be easily related to search or combinatorial optimization problems. They are all NP-complete problems which show a special kind of second-order phase transition, known as algorithmic transitions, in the context of the computational resources required to solve the problem (6), typically time. This computational cost is largely independent of implementation details as it is a direct measure of the algorithmic complexity of the problem.

In these algorithmic phase transitions, the constraints of the system act as the control parameter. In general, different behaviours are found on both sides of the critical point. In the phase with many constraints, the order parameter hardly changes, and in the phase with few constraints, the order parameter changes gradually as one moves away from the critical point. In the critical region, varying the control parameter leads to abrupt changes in the typical complexity, similar to the abrupt changes in phase transitions in physical systems.

All these optimisation problems present an easy-hard-easy pattern that appears as a consequence of a trade-off between the number of solutions and the size of the search space, with a maximum of hardness around the critical point, as the number of constraints varies (7): when the system constraints are very high, it is easy for the algorithm to find a solution because the search space is small, and the system finds a solution quickly. As constraints decrease, the search space grows and the computational cost increases until it reaches a maximum. From there, constraints keep decreasing, but now the number of available solutions grows exponentially. It becomes increasingly easier for the system to find a solution.

In the context of our system, the coding sequence (CDS) phase should correspond to the phase with more constraints, as the order parameter ρ does not change in this phase. Accordingly, the nCDS phase should correspond to the phase with less constraints, and as would be expected, here the order increases abruptly, growing continuously as $\langle L \rangle$ grows, and the system obtains complexity "for free" by means of the transition.

Consequently, as we move from the CDS into the nCDS phases, the constraints should decrease. This is exactly what we find: for a mean gene length $\langle L \rangle$, the size of the search space is proportional to $4^{\langle L \rangle}$. Thus, the smaller $\langle L \rangle$ is, the smaller (more constrained) the search space is. The latter is the case of the CDS phase, characterized by small $\langle L \rangle$'s. As $\langle L \rangle$ increases and the system enters in the nCDS phase, the search space increases and the system becomes less constrained. Therefore $\langle L \rangle$ (or better $1/\langle L \rangle$) is a good proxy of the constraints of the system.

The evolutionary search algorithm finds solutions by expressing certain molecules, RNA, and proteins. In the CDS phase, the algorithm has a linear behaviour: a gene of length l generates a protein of length $l/3$. But finding a protein of a given length is an NP-hard problem (8), which implies that the corresponding gene must explore a space of size 4^{l}. Thus, as the search space grows exponentially with the mean length, the problem tends to be harder. On the other hand, in the nCDS phase, the algorithm has an exponential behaviour: now a single gene can generate several proteins thanks to alternative splicing, increasing the number of possible solutions, and also free RNAs, which now have metabolic and regulatory functions. The system's available solutions grow exponentially, thus offsetting the growth of the search space.

From this perspective, we can understand evolution as a search algorithm that finds, in the space of possible genes, a solution of mean gene length $\langle L \rangle$ in time t. But since this mean gene length does not grow linearly with time but rather exponentially, the mean processing speed $v(t)$ changes over time following the equation:

$$v(t) = \frac{\langle L(t) \rangle}{t} = \frac{L_0 \cdot \exp\left((\langle \zeta \rangle - 1) \cdot t\right)}{t}.$$

In the context of an easy-hard-easy transition, this processing speed should decrease until it becomes minimal at a critical value t_c, where the algorithm presents its greatest inefficiency, and grow again from that moment on. Note that the algorithmic complexity is usually proportional to time and constraints. As we can approximate constraints by the inverse of the mean gene

length, following ref. 9, we can then define an approximation to the algorithmic complexity for our system as the inverse of this speed:

$$\tau(t) = \frac{t}{\langle L(t)\rangle}, \qquad [14]$$

that is, the average time taken per base to reach a solution of mean gene length $\langle L(t)\rangle$.

Solving for time from Eq. (1), we obtain:

$$t(\langle L\rangle) = \frac{1}{\langle\zeta\rangle - 1}\log\left(\frac{\langle L\rangle}{L_0}\right), \qquad [15]$$

so that the algorithmic complexity as a function of the mean gene length is:

$$\tau\left(\langle L\rangle\right) = \frac{\log\left(\frac{\langle L\rangle}{L_0}\right)}{\left(\langle\zeta\rangle - 1\right)\langle L\rangle}.$$

If we assume that we are facing an algorithmic transition, this function should have a maximum and it should coincide with L_c. Therefore:

$$\left.\frac{d\tau}{d\langle L\rangle}\right|_{\langle L\rangle = L_c} = \left.\frac{\left(\langle\zeta\rangle - 1\right)\left(1 - \log\left(\langle L\rangle / L_0\right)\right)}{\left(\left(\langle\zeta\rangle - 1\right)\langle L\rangle\right)^2}\right|_{\langle L\rangle = L_c} = 0,$$

which leads to the following solution for the critical point:

$$L_c = e \cdot L_0. \qquad [16]$$

Using our previous estimate of $L_0 = 554$, we find an estimate for the mean critical length: $L_c = e \cdot 554 = 1,506$, a value very close to what we had previously estimated from the data: $L_c = 1,500$. In Fig. 4 (inset), we have plotted as coloured dots the experimental algorithmic complexity $\tau(t)$, proving that it shows a typical easy-hard-easy pattern with the maximum coinciding with this theoretical prediction, very close to the critical point.

The value of L_0 in Eq. (16) determines the dependence of L_c on Taylor's law. Indeed, L_0, represents the mean gene length of LUCA's genome, the simplest solution that determines the minimum algorithmic complexity in our system. We can solve for L_0 from Eq. (10), so that:

$$L_0 = \left(a\exp\left(2G_0\right)\right)^{\frac{1}{4-\beta}},$$

and therefore

$$L_c = e \cdot \left(a\exp\left(2G_0\right)\right)^{\frac{1}{4-\beta}}.$$

Finally, if Eq. (16) is right, then the critical time t_c, the moment at which the critical length is reached, will be according to Eq. (15):

$$t_c = t\left(L_c\right) = \frac{1}{\langle\zeta\rangle - 1}. \qquad [17]$$

According to ref. 10, approximately 3,600 My have gone by since the emergence of LUCA at $t = 0$ (measured as the divergence time between bacteria and eukaryotes). The chronology from the first eukaryotic common ancestor (FECA) to the last eukaryotic common ancestor (LECA) is estimated to be around $(2900, 2300)$ My ago (11–16), thus FECA is estimated to have emerged at $t = 3,600 - 2,900 = 700$ and LECA at $t = 3,600 - 2,300 = 1,300$ My after LUCA. Using our estimate $\langle\zeta\rangle - 1 = 0.00101$, we have that the critical time is: $t_c = t\left(L_c\right) = 990$ My after LUCA, which is located in the middle of the fork $(700, 1300)$. Observe that if we use instead $L_c = 1,500$ and $L_0 = 554$, from Eq. (15) we obtain: $t_c = 986$ My after LUCA, a similar value. More generally, we have performed a sensibility analysis to check to what degree our estimate of t_c depends on the exact value of L_c as this figure comes from an estimation from the data in Fig. 3. Specifically, we have varied the value of L_c in the range (1300, 1700). For this range and using Eq. (16) with $L_0 = 554$, we obtain:

$$t_c = \frac{1}{0.00101}\log\left(\frac{L_c}{554}\right), \qquad [18]$$

which returns $t_c \in (844, 1110)$, a figure well within the FECA-to-LECA interval (700, 1300).

The values of t_c are specially sensitive to the value $\langle\zeta\rangle - 1$. Using a range for $\langle\zeta\rangle - 1 \in (0.0008, 0.0012)$ in:

$$t_c = \frac{1}{\langle\zeta\rangle - 1}\log\left(\frac{1,500}{554}\right), \qquad [19]$$

we obtain $t_c \in (830, 1245)$, again within the FECA-to-LECA interval.

And combining both uncertainties, taking the intervals $L_c \in$(1,300, 1700) and $\langle\zeta\rangle - 1 \in (0.0008, 0.0012)$, in:

$$t_c = \frac{1}{\langle\zeta\rangle - 1}\log\left(\frac{L_c}{554}\right), \qquad [20]$$

we obtain $t_c \in (711, 1401)$, practically coinciding with the interval.

Therefore, by assuming that the transition is algorithmic, we are able to account for the dependence of the transition (equation 4 of main text) on Taylor's law, and determine both the critical length—which is close to 1,500 base pairs—and the critical time—which coincides with the estimated origin of the first eukaryotes.

12. Figures

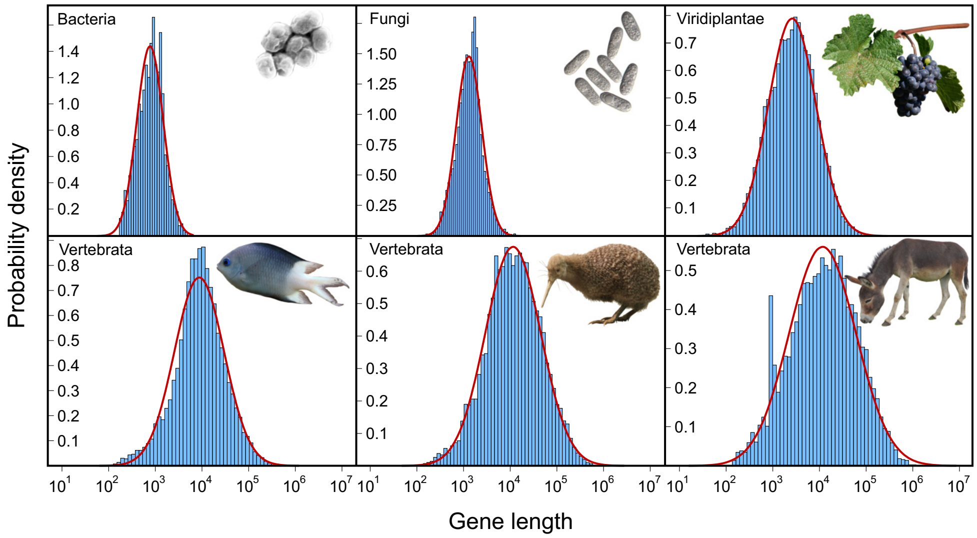

Fig. S1. Lognormalcy across the tree of life. Histograms of gene length for a bacterium (*Acinetobacter baumannii*), a fungus (*Colletotrichum gloeosporioides*), a plant (*Vitis vinifera*), a fish (*Acanthochromis polyacanthus*), a bird (*Apteryx owenii*), and a mammal (*Equus asinus asinus*). Gene length is measured in number of base pairs and is represented on a logarithmic scale. In the case of non-fish vertebrates, it is rather frequent the presence of a peak corresponding to sense receptors, as seen in the sixth histogram. In the vast majority of organisms, the gene length distribution is lognormal. Only a handful of histograms show odd shapes such as two maxima or a noisy distribution.

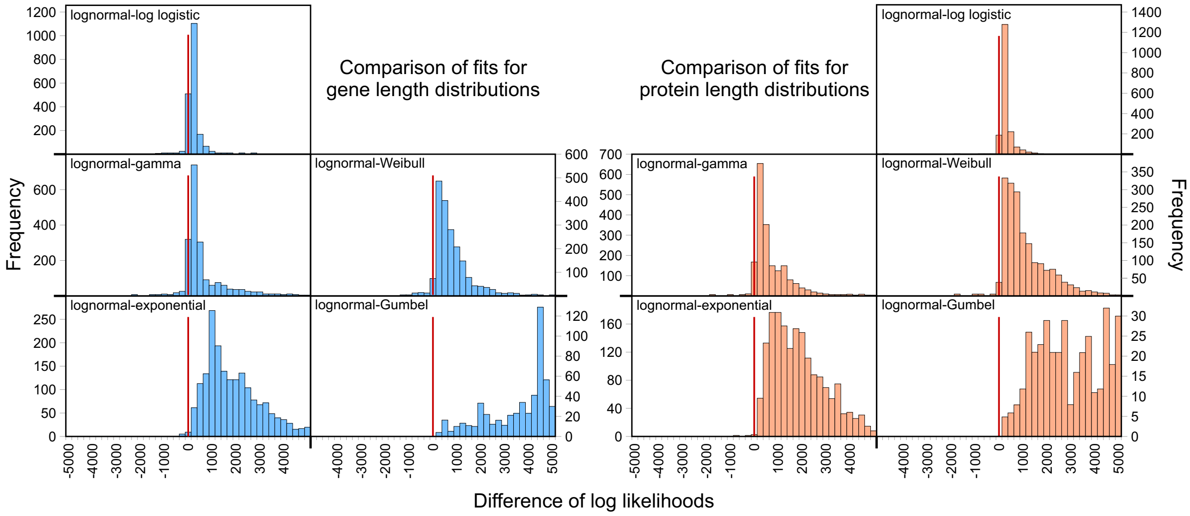

Fig. S2. The lognormal distribution fits gene and protein length data better than alternative distributions. Left: histograms for the difference of log-likelihoods for the best fit of gene length distributions to a lognormal distribution and to a competing distribution. The vertical red lines, corresponding to 0, indicate cases when the best fit for both candidate distributions are comparable. Cases to the right of this red line, i.e., cases with positive differences, indicate that the lognormal distribution fits better than the alternative distribution. Conversely, cases to the left of the red line indicate that the alternative distribution fits better than the lognormal distribution. Right: histograms for the difference of log-likelihoods for the best fit of protein length distributions to a lognormal distribution and to a competing distribution. In both cases, the lognormal distribution provides the best fits, followed by the log logistic, gamma, Weibull, exponential, and Gumbel distributions in decreasing order.

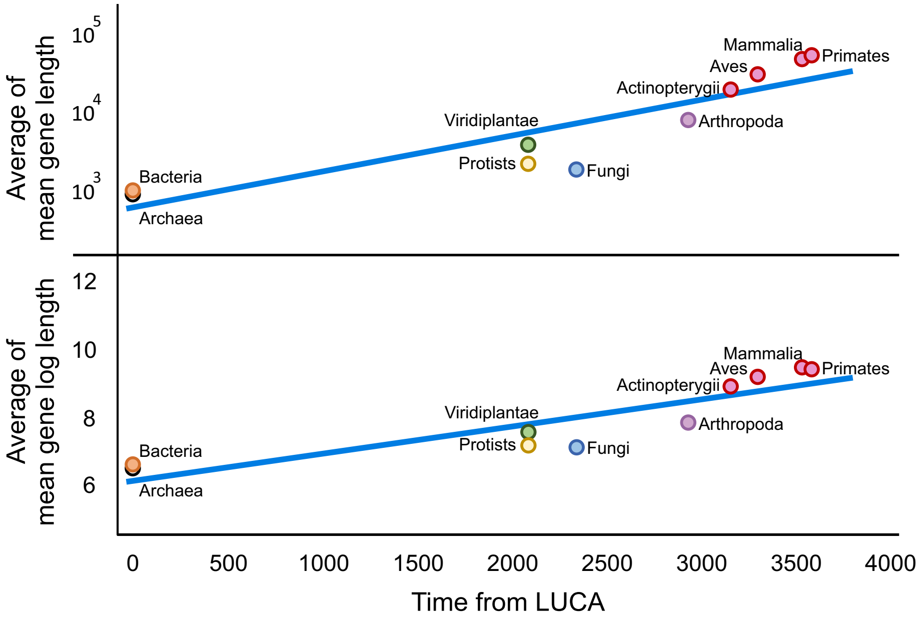

Fig. S3. Gene growth along time. For the abscissa, taking *Homo sapiens* as a reference, we assigned median divergence times to each phyletic group using Timetree of Life v5 (Materials and Methods), and converted them to times from LUCA assuming LUCA existed $3,600$ million years ago (Timetree of Life v5; Materials and Methods). For the ordinate, we have averaged the mean gene lengths (and mean gene log lengths) for all the organisms in a given group and assigned this average to the group (Materials and Methods). The result shows that mean gene length (top) and mean gene log length (bottom) have increased across evolution following an exponential, and a linear growth, respectively. The blue lines show an exponential (top) and a linear (bottom) fit, resulting in $e^{6.3(\pm 0.5)+0.00106(\pm 0.00019)t}$ and $6.2(\pm 0.4) + 0.00080(\pm 0.00014)t$ respectively, with a goodness of fit $R^2 = 0.79$ in both cases. Time is expressed in million years and gene length is represented in number of base pairs. Here, Mammalia refers to non-primate Mammalia.

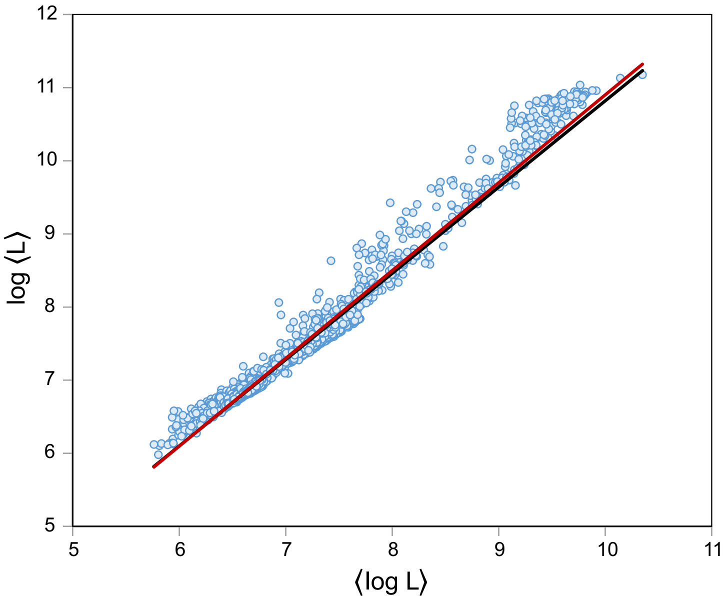

Fig. S4. The linear relationship between the logarithm of the mean, and the mean of the logarithm for gene lengths. Blue dots represent data from the gene length distributions, and the red line denotes its linear fit ($R^2 = 0.97$): $\log \langle L(t) \rangle = 1.2013(\pm 0.0012) \cdot \langle \log L(t) \rangle - 1.112(\pm 0.008)$. The black line, which is virtually the same, illustrates the theoretical linear relationship, $\log \langle L(t) \rangle = 1.16 \cdot \langle \log L(t) \rangle - 0.86$, deduced from the mean and logarithm mean growth laws and Taylor's law (equation 3 in SI) using our improved estimation of parameters on page 6 of SI. Gene length is represented in number of base pairs.

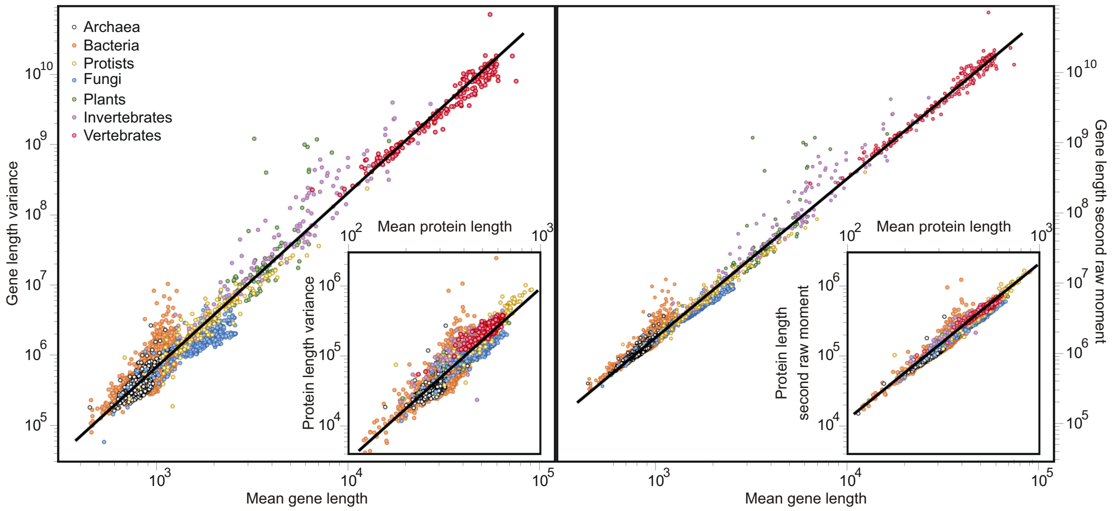

Fig. S5. Power laws for the variance and for the second moment. Power law for the variance (left) and for the second raw moment (right) vs mean length; main graphs are for gene lengths (in number of base pairs) and insets for protein lengths (in number of amino acids). The standard Taylor's law (left) is the asymptotic approximation of the generalised Taylor's law (right). The latter shows a smaller dispersion of data and provides a better fit (black lines): left, $0.0159(\pm 0.0004)\langle L\rangle^{2.511(\pm 0.004)}$, $R^2 = 0.92$ for genes and $0.0168(\pm 0.0015)\langle L_p\rangle^{2.605(\pm 0.016)}$, $R^2 = 0.73$) for proteins (inset); right, $0.208(\pm 0.003)\langle L\rangle^{2.292(\pm 0.002)}$, $R^2 = 0.98$ for genes and $0.388(\pm 0.015)\langle L_p\rangle^{2.241(\pm 0.007)}$, $R^2 = 0.73$ for proteins (inset). The values of the parameters from the fit of the second raw moment are the ones that one should take as valid, since the graphs on the left correspond to asymptotic approximations which overestimate the exponent and underestimate the coefficient.

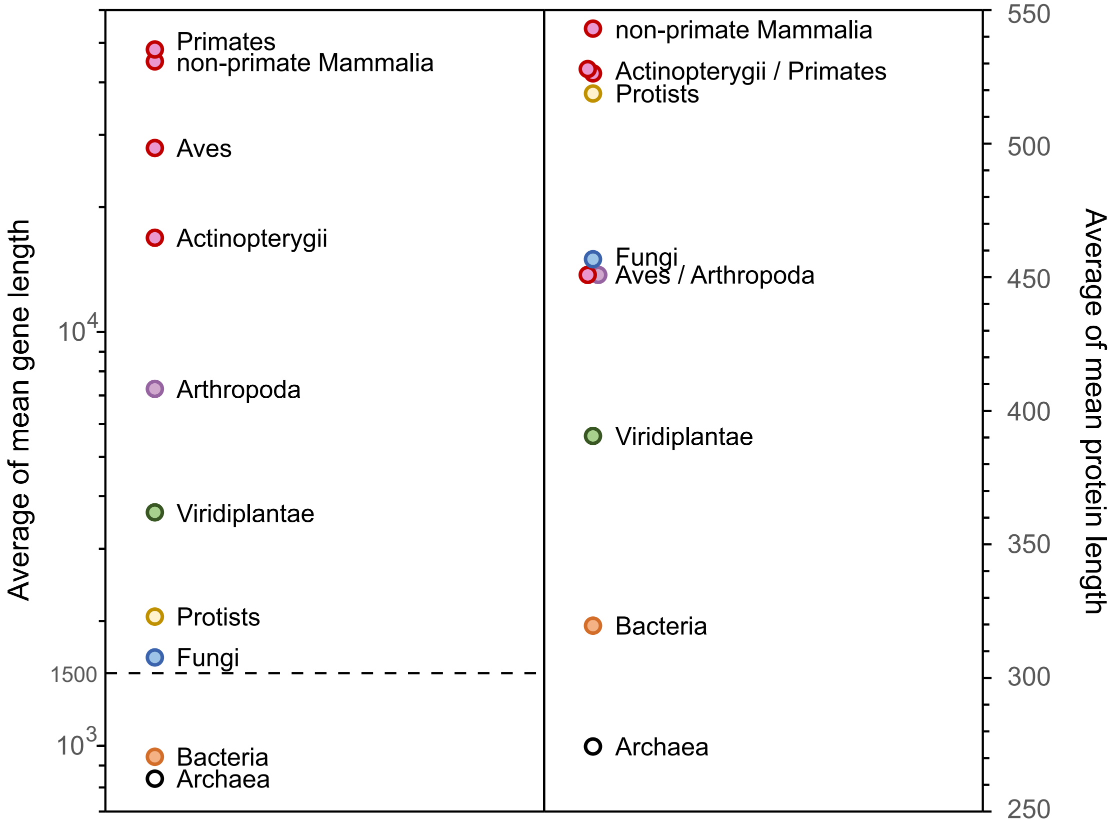

Fig. S6. Mean gene length vs mean protein length. The figure shows the average of mean gene lengths (left, in number of base pairs) and mean protein lengths (right, in number of amino acids) for several phyletic groups. Mean gene and protein lengths have been averaged across all the organisms within a group. This average, then, has been assigned to the entire group (Materials and Methods). This procedure minimises noise and the statistical particularities that a certain organism could have. As noted, mean gene length is a better proxy for organismal complexity than mean protein length.

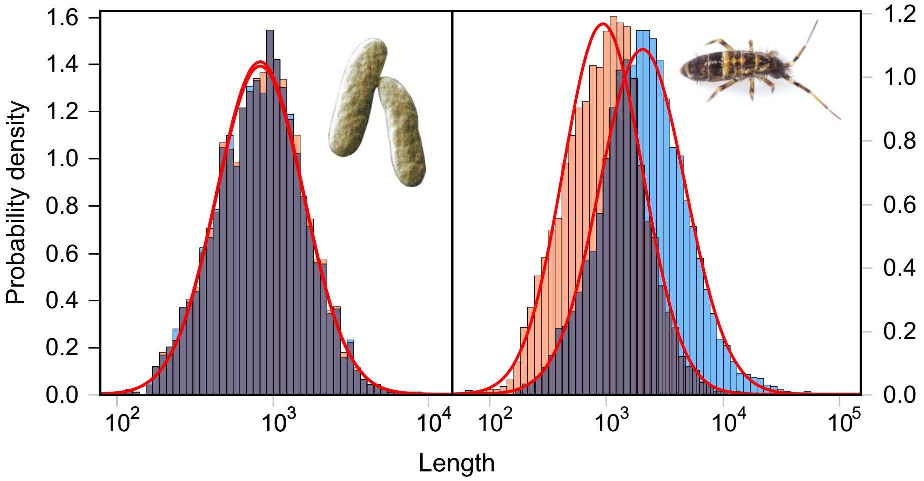

Fig. S7. Rescaling protein distributions. Histograms of gene lengths (blue) and 3 times protein lengths (orange) so that both distributions are in the same units, i.e., number of base pairs. Note that lengths are represented on a logarithmic scale. Left and right panels display data for a prokaryote (the bacterium *Shewanella loihica*), and the insect *Orchesella cincta*, respectively. In prokaryotes, this rescaling leads to a match between the distributions of protein and gene lengths. This is not the case for eukaryotes due to the presence of gene non-coding sequences.

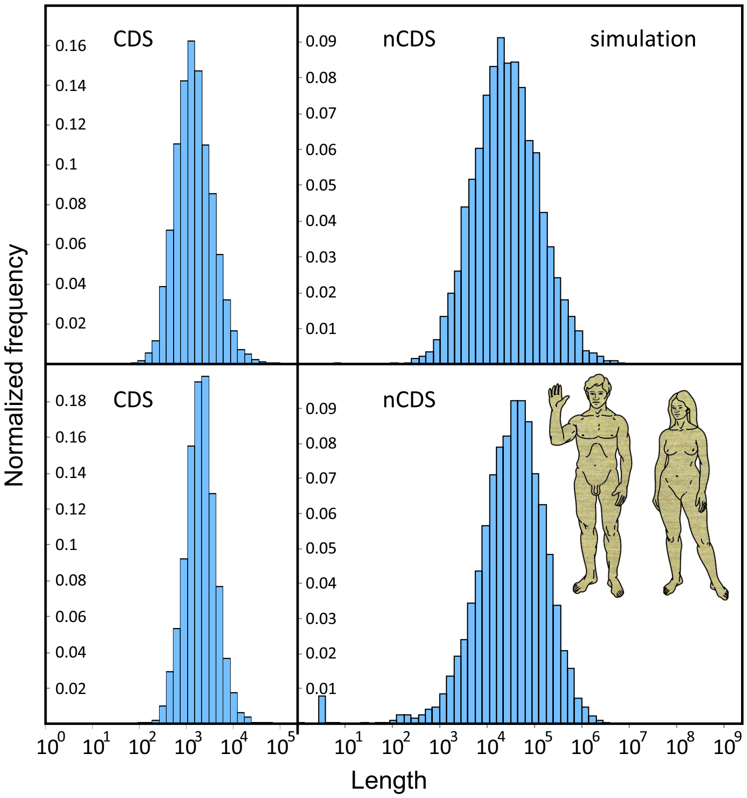

Fig. S8. Distributions for coding (CDS) and non-coding (nCDS) intragenic sequences. Top: outcome of a simulation of gene growth started with a distribution typical of a bacterium and stopped when the mean gene length matches that of modern humans. When the mean length of the simulation crosses the critical length $Lc = 1,500$, simulated genes grow only by increasing nCDS (Materials and Methods). Bottom: distributions of CDS and nCDS for *Homo sapiens* (Materials and Methods). Gene length is represented in number of base pairs and is plotted on a logarithmic scale.

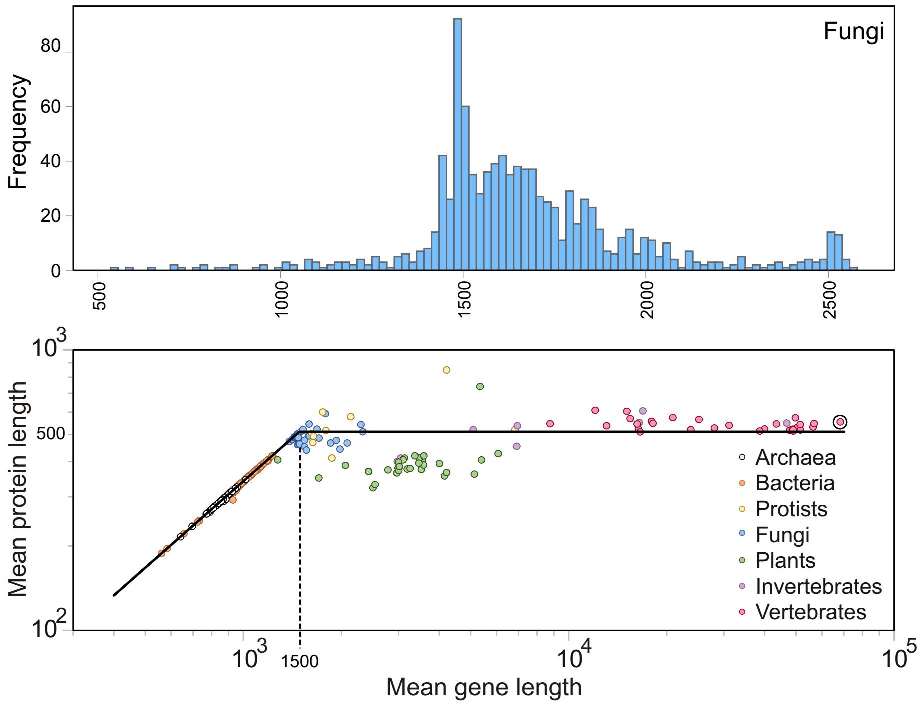

Fig. S9. The threshold at 1,500 base pairs. Top: histogram of mean gene lengths for 1,014 fungi. The figure shows a conspicuous sharp peak at 1,500 base pairs, indicative of the existence of critical slowing down around this value. Bottom: threshold in the relationship between mean protein and gene lengths as in Fig. 3 in main text, but only for the best annotated genomes. This figure shows more clearly that the change in the trend occurs at 1,500 base pairs. Protein length is measured in number of amino acids.

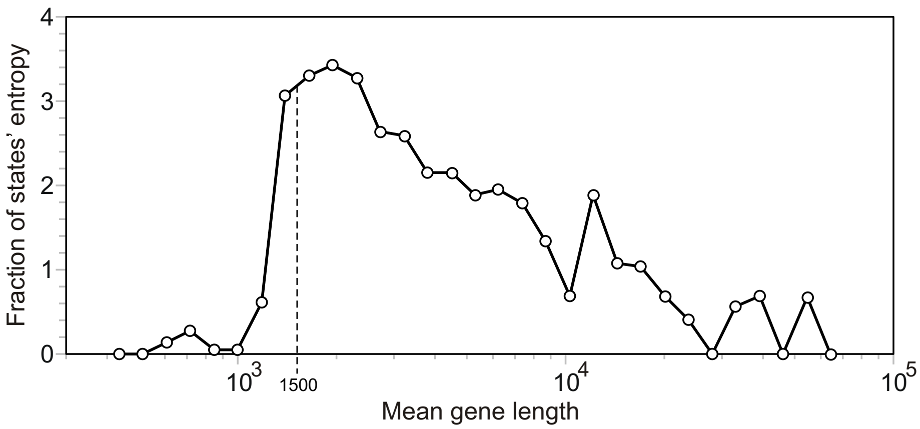

Fig. S10. Entropy of the scatter of values for the fraction of gene non-coding sequences (i.e., fraction of states) at a given mean gene length shown in Fig. 4 of main text. Gene length is measured in number of base pairs.

13. SI References

1. Giometto A, Formentin M, Rinaldo A, Cohen JE, Maritan A (2015) Sample and population exponents of generalized taylor's law. *Proc. Natl. Acad. Sci. U.S.A.* 112(25):7755–7760.
2. Nevers Y, Glover NM, Dessimoz C, Lecompte O (2023) Protein length distribution is remarkably uniform across the tree of life. *Genome Biol.* 24(1):135.
3. Bunde A, Havlin S (1991) *Percolation II.* (Springer Berlin Heidelberg, Berlin, Heidelberg), pp. 97–150.
4. Kirpatrick S, Selman B (1994) Critical behavior in the satisfiability of random boolean expressions. *Science* 264(5163):1297–1301.
5. Hogg T, Huberman BA, Williams CP (1996) Phase transitions and the search problem. *Artif. Intell.* 81(1):1–15. Frontiers in Problem Solving: Phase Transitions and Complexity.
6. Moore C, Mertens S (2011) *The Nature of Computation.* (Oxford University Press), p. 985.
7. Percus A, Istrate G, Moore C (2005) *Computational Complexity and Statistical Physics.* (Oxford University Press).
8. Pierce NA, Winfree E (2002) Protein design is np-hard. *Protein Eng.* 15(10):779–782.
9. Lacasa L, Luque B, Miramontes O (2008) Phase transition and computational complexity in a stochastic prime number generator. *New J. Phys.* 10(2):023009.
10. Kumar S, et al. (2022) TimeTree 5: An Expanded Resource for Species Divergence Times. *Mol. Biol. Evol.* 39:msac174.
11. Retallack GJ, Krull ES, Thackray GD, Parkinson D (2013) Problematic urn-shaped fossils from a paleoproterozoic (2.2 ga) paleosol in south africa. *Precambrian Res.* 235:71–87.
12. Albani AE, et al. (2010) Large colonial organisms with coordinated growth in oxygenated environments 2.1 gyr ago. *Nature* 466(7302):100–104.
13. Buick R (2010) Ancient acritarchs. *Nature* 463(7283):885–886.
14. Etter W (2013) *Patterns of diversification and extinction.* (Springer-Verlag, Berlin, Heidelberg), pp. 1–60.
15. Chernikova D, Motamedi S, Csürös M, Koonin EV, Rogozin IB (2011) A late origin of the extant eukaryotic diversity: divergence time estimates using rare genomic changes. *Biol. Direct* 6:1–18.
16. Dacks JB, et al. (2016) The changing view of eukaryogenesis–fossils, cells, lineages and how they all come together. *J. Cell Sci.* 129(20):3695–3703.
